# Structure of Ni(II) Inclusion Complex in Solid/Solution States and the Enhancement of Catalytic Behavior in Electrochemical Hydrogen Production

**DOI:** 10.3390/molecules29245858

**Published:** 2024-12-12

**Authors:** Tomohiko Hamaguchi, Yuudai Iseki, Ryuta Ishikawa, Akio Mishima, Satoshi Kawata

**Affiliations:** Department of Chemistry, Faculty of Science, Fukuoka University, 8-19-1 Nanakuma, Jonan-ku, Fukuoka 814-0180, Japanryutaishikawa@fukuoka-u.ac.jp (R.I.); akio.mishima@fukuoka-u.ac.jp (A.M.); kawata@fukuoka-u.ac.jp (S.K.)

**Keywords:** Ni complex, cyclodextrin, inclusion, electrochemistry, hydrogen evolution reaction

## Abstract

In this article, we investigate the encapsulation of K_2_[Ni(maleonitriledithiolate)_2_] (**1**) within a host molecule, β-cyclodextrin (β-CD), via single-crystal X-ray analysis. An inclusion complex, K_2_{[Ni(maleonitriledithiolate)_2_]@(β-CD)_2_} (**2**), was constructed from **1** and two β-CDs. The anion guest Ni complex included a host cavity, constructed using two β-CDs, and the Ni atom of the anion was located between the two hydrophilic primary rims. Ultraviolet-visible absorption spectroscopy revealed that inclusion complex **2** exhibited a 2:1 (host:guest) stoichiometry in the solution, which is consistent with the result obtained from X-ray crystallography. The association of the host and guest occurred in two steps, and the association constants for the first and second steps were 1.1(7) × 10^4^ and 1.8(5) × 10^4^ mol^−1^ dm^3^, respectively. The catalytic behavior of **1** and **2** was investigated for electrochemical hydrogen production in the aqueous solution of an acetate buffer (pH = 4.72). During the catalytic reaction, inclusion complex **2** was observed to have a better catalytic reaction rate than **1**. The study findings provide insights into the effects of the encapsulation of guest molecules within host structures.

## 1. Introduction

The encapsulation of guest molecules in host structures can enable alterations in the chemical reactivity of molecules, offering a valuable opportunity in the fields of chemistry and material science. Furthermore, encapsulation can regulate the solubility, absorption, emission, and electrochemical properties of guest molecules [1,2,3,4]. The production of artificial metalloenzymes is a unique approach to constructing catalysts using a protein as a host and an abiotic metal cofactor as a guest [5,6,7]. Recently, the demand for sustainable energy has increased, and hydrogen molecules have become attractive as alternatives to the current use of fossil fuel energy [8]. In nature, hydrogenases are high-performance catalysts for hydrogen molecules; therefore, many scientists have attempted to establish an effective catalyst inspired by hydrogenase [9,10,11].

**Figure 1:**
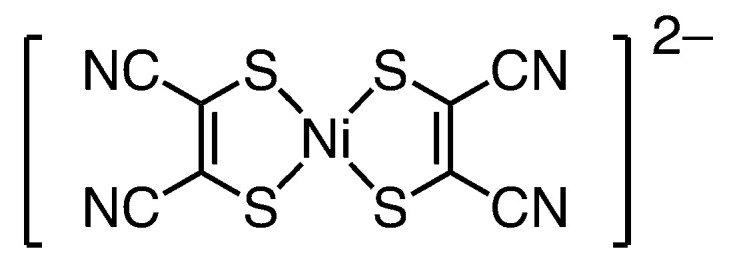
Molecular structure of an anion of the free Ni complex **1**.

Cyclodextrins (CDs), which are well-known host molecules, are a class of cyclic oligosaccharides comprising α-1,4-linked glucopyranose units [12]. These unique ring-shaped molecules feature a hydrophobic central cavity and hydrophilic exterior, allowing them to form inclusion complexes with various guest molecules. Such inclusion complexes have been reported for their structure and the regulation of chemical reactions and properties [13,14]. Darensbourg et al. reported an inclusion complex formed by a small-molecule model complex of the [FeFe]-H_2_ase active site and β-CD and investigated its electrochemical catalytic behavior [15]. They concluded that the inclusion complex exhibited catalytic behavior during electrochemical hydrogen production and that proton reduction occurred at a more negative potential. This reflected the hydrophobic environment of the cavity of β-CDs compared with a small-molecule model complex without β-CDs. We have previously reported the crystal structure of an inclusion complex, {[Cu(2-pyridinemethanolate)(2-pyridinemethanol)]_2_@(γ-CD)_2_}(PF_6_)_2_ [16], constructed using two γ-CDs as a host and [Cu(2-pyridinemethanolate)(2-pyridinemethanol)]^−^ as a guest. The Cu ions were located between the two hydrophilic secondary rims. Thus, a similar square-planner complex could regulate catalytic behavior by encapsulation within CDs. Ni et al. reported the electrochemical hydrogen production and catalytic behavior of [Ni^II^(mnt)_2_]^2−^ and [Ni^III^(mnt)_2_]^−^ complexes in aqueous and acetic acid/acetonitrile solutions with various acidity levels (mnt = maleonitriledithiolate) [17,18]. Furthermore, Meng et al. revealed that the [Ni^III^(mnt)_2_]^−^ complex can form an inclusion complex with β-CD in the solid and solution states [19]. Thus, we investigated the K_2_[Ni^II^(mnt)_2_] complex (**1**) (Figure 1) for the present study.

Herein, we report an inclusion complex, K_2_{[Ni(mnt)_2_]@(β-CD)_2_} (**2**); its structure was investigated via X-ray crystallography and absorption spectroscopy. Furthermore, we compared its catalytic behavior with that of the free Ni complex **1** to evaluate the effect of encapsulation. The study findings highlight the relationship between the encapsulation of guest molecules within host structures and their catalytic behavior.

## 2. Results and Discussion

### 2.1. Molecular Structure in the Solid Form

**Figure 2:**
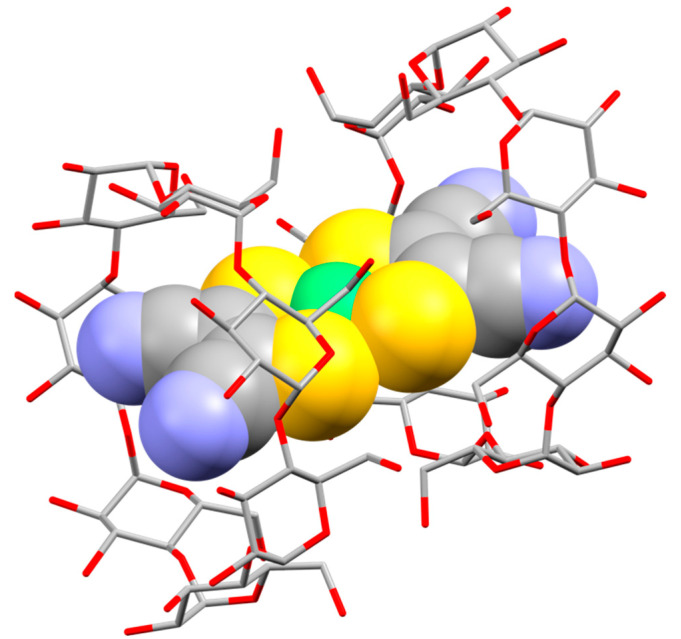
Molecular structure of the anion of inclusion complex **2** using a CPK model ([Ni(mnt)_2_]^2−^) and capped sticks model (β-CD). Light green, Ni; yellow, S; gray, C; blue, N; red, O. Counter cations and water molecules are omitted for clarity. Selected bond distances and angles: Ni1–S1 = 2.176(3) A°, Ni1–S2 = 2.162(2) A°, S1–Ni1–S2 = 91.69(8)°, S1–Ni1–S1* = 88.25(16)°, S2–Ni1–S2* = 88.79(12)°, S1*–Ni1–S2 = 175.08(17)°. * indicates the equivalent atoms generated by the symmetry operator (−x + 1, y, −z).

**Figure 3:**
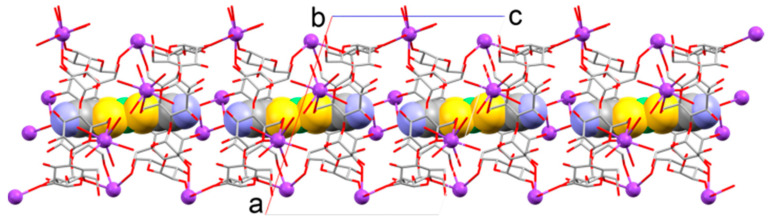
Molecular channels in the crystal of inclusion complex **2** along the c-axis (viewed from the b-axis).

Figure 2 and Figure S1 show the crystal structure of inclusion complex **2**, which is crystallized in the chiral space group *C*2. The crystal comprised one anion guest complex [Ni(mnt)_2_]^2−^, two potassium counter cations, two β-CDs, and 20 crystallization water molecules. The dihedral angle between the S1–Ni1–S2 and S1*–Ni1–S2* planes (6.8(2)°) indicates that the anion guest complex exhibited a slightly distorted square-planar geometry (* indicates the equivalent atoms generated by the symmetry operators (−x + 1, y, −z). The numbering scheme is shown in Figure S2). The anion guest complex included a host cavity constructed using two β-CDs via direct hydrogen bonding between their primary rims, as well as hydrogen bonding between their primary rims through solvent water molecules and potassium counter cations. The Ni atom of the anion guest complex was located between the two hydrophilic primary rims. Figure 3 and Figure S3 show the packing patterns of the inclusion complex, viewed from the b- and c-axes, respectively. The inclusion complex was connected to neighbor inclusion complexes via direct hydrogen bonding between their secondary rims and hydrogen bonding between their secondary rims through solvent water molecules and potassium counter cations, resulting in a molecular channel along the c-axis. In addition, the molecular channels were connected to neighbor molecular channels (Figure S3). Meng et al. reported the crystal structure of an inclusion complex constructed using [Ni^III^(mnt)_2_]^−^ and β-CD [19]. Their complex generally exhibited almost the same structure as that of the present inclusion complex. For example, both complexes possessed an encapsulated [Ni(mnt)_2_]^n−^ in a cavity of two β-CDs. The cavity was constructed via the interaction between the primary rims of β-CDs, and the inclusion complexes contained molecular channels. However, a notable difference is that the inclusion complex reported by Meng et al. had one “empty” β-CD.

The [Ni(mnt)_2_]^n−^ complex can be generally isolated as Ni(II) and Ni(III). The presence of two counter cations in the inclusion complex implies that the oxidation number of Ni in the inclusion complex could be two. However, the disorder of the counter potassium cation obscures the number of cations. Oxidation number will be discussed in the section describing ^1^H NMR results (see Section 2.2 on molecular structure in the solution form).

To evaluate the inclusion of the anion guest complex [Ni(mnt)_2_]^2−^ into β-CD, an independent gradient model based on the Hirshfeld partition of molecular density (IGMH) method was used to visualize the interaction between β-CD and the free Ni complex [20]. As shown in Figure S4, there are some van der Waals interactions (green isosurfaces) between the β-CDs and cyano groups of the anion guest complex. These interactions would stabilize the inclusion. To estimate the interaction in the solid form, the IR spectrum was evaluated (Figure S5). The prominent IR peaks of the free Ni complex were *ν*_max_ = 2208 and 2198 cm^−1^, which can be attributed to *ν*(CN). The corresponding peak of inclusion complex **2** was *ν*_max_ = 2198 cm^−1^. The result appears to show that the *ν*(CN) of the inclusion complex indicated a redshift compared with that of the free Ni complex. The redshift would be caused by the interaction. The shift was tiny; however, quantum calculations revealed that the shift was not negligible (Figure S6; see also Section 3.4).

### 2.2. Molecular Structure in the Solution Form

An encapsulation in solution form was investigated with ^1^H NMR in a D_2_O solution (Figures S7 and S8). Inclusion complex **2** exhibited signals at *δ* = 3–5 ppm (Figure S7). Compared with those of β-CD (Figure S8), the signals of the inclusion complex exhibited similar moderate sharpness within the typical range of chemical shifts. This observation indicates that the inclusion complex contains a diamagnetic Ni center in the anion guest complex [Ni(mnt)_2_]^2−^. This result was further supported by the Ni–S bond distances (Figure S9). The NMR spectra of β-CD and the inclusion complex differed. This difference implies that the anion guest complex would interact with β-CD, even in solution form, as has been reported elsewhere [15,19,21]. ^13^C NMR was also measured to investigate the inclusion from the signals of the anion guest complex (Figures S10 and S11). We measured the NMR spectrum at almost the same concentration as that used in the absorption spectral and electrochemical study, and the signals of the guest were not detected.

**Figure 4:**
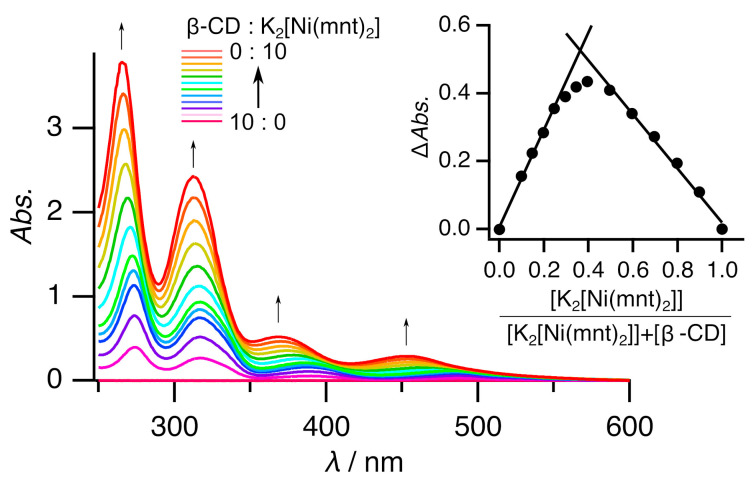
UV-Vis spectra of the various ratios of free Ni complex **1** vs. β-CD in water. [K_2_[Ni(mnt)_2_]] + [β-CD] = 1 × 10^−3^ mol dm^−3^. The inset shows the Job’s plot at 278 nm. Thin arrows indicate spectral changes.

**Figure 5:**
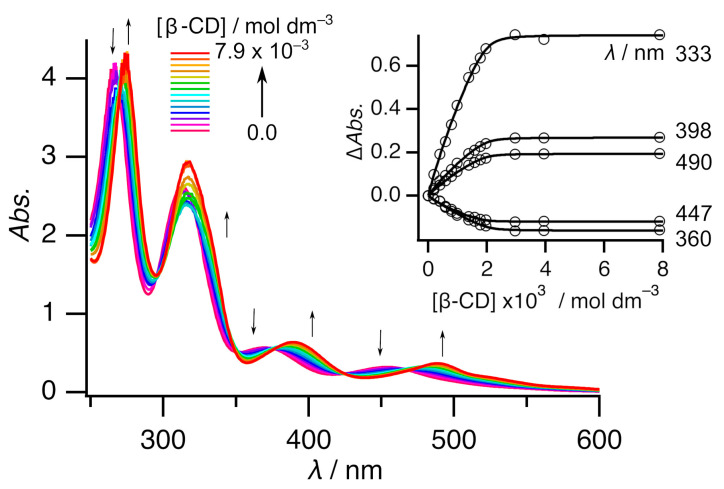
UV-Vis spectra of free Ni complex **1** with various amounts of β-CD in a 1 mol dm^−3^ Na_2_SO_4_ aqueous solution. [K_2_[Ni(mnt)_2_]] = 1 × 10^−3^ mol dm^−3^. Thin arrows indicate spectral changes. The inset shows the curve fitting of plot Δ*Abs.* vs. [β-CD] at five points.

The stoichiometry of inclusion complex **2** in solution form was calculated using the continuous variation method (Job’s plot) via UV-Vis absorption spectroscopy. The results are shown in Figure 4. The inset in Figure 4 shows that the analysis at 278 nm resulted in a maximum at 0.36, indicating that the host:guest ratio was 2:1 in the solution. This ratio was consistent with the X-ray crystallography result. Thus, the molecular structure in solution form would be the same as that in solid form. Titration was performed to elucidate the association (Figure 5, Figures S12, and S14). The free Ni complex exhibited four peaks at ca. 265, 313, 370, and 453 nm. All the peaks exhibited redshifts with each addition of β-CD, eventually reaching ca. 274, 317, 389, and 489 nm, respectively. These shifts were consistent with those in a previous report on [Ni^III^(mnt)_2_]^−^ with β-CD [19]. In addition, this finding was predicted by quantum calculations (Figure S15; see also Section 3.4). These shifts could be caused by (a) the structural change from a square-planar geometry to the slightly distorted square-planar geometry and/or (b) the difference in solvation between that in hydrophilic aqueous solution and that in hydrophobic β-CD. Figure S16 shows a comparison of the calculated [Ni^II^(mnt)_2_]^−^ spectra between the square-planar geometry and the slightly distorted square-planar geometry. It is interesting that the slight distortion results in little difference in the calculated spectra. In contrast, Figure S17 shows a comparison between its behavior in water and that in heptane. In this comparison, the spectrum in hydrophobic heptane shows a bathochromic shift rather than that seen in the spectrum in hydrophilic water. Therefore, the redshift of peaks upon titration is caused by the inclusion.

A more detailed analysis of the titration revealed that this association could be observed in two steps. The first step involved adding β-CD (0–1 eq.); the spectrum continuously changed, with seven isosbestic points (270, 295, 322, 352, 376, 424, and 467 nm) (Figure S12). The second step involved adding β-CD (1–2 eq.); the spectrum continuously changed, with six isosbestic points (269, 299, 348, 376, 426, and 472 nm) (Figure S13). The spectrum was almost the same, even when more than 2 eq. of β-CD was added (Figure S14). Thus, two successive association reactions were assumed, and two association constants were defined, as below.

(1) $$K_{2}\left[Ni(mnt{)}_{2}\right]+\beta \text{-}CD\;\overset{K_{1}}{⇌}K_{2}\{[Ni(mnt{)}_{2}]@(\beta \text{-}CD)\}$$

(2) $$K_{2}\{\left[Ni(mnt{)}_{2}\right]@\left(\beta \text{-}CD\right)\}+\beta \text{-}CD\;\overset{K_{2}}{⇌}K_{2}\{[Ni(mnt{)}_{2}]@(\beta \text{-}CD{)}_{2}\}$$

Curve fitting of plot Δ*Abs.* vs. [β-CD] at five points was conducted (see the inset in Figure 5); the fitting estimates for *K*_1_ and *K*_2_ were 1.1(7) × 10^4^ and 1.8(5) × 10^4^ mol^−1^ dm^3^, respectively. These values suggest that inclusion complex **2** remained at 80% in a solution of [complex **2**]_0_ = 5 × 10^−4^ mol dm^−3^ and 87% in a solution of [complex **2**]_0_ = 1 × 10^−3^ mol dm^−3^ [22]. Thus, we can investigate the inclusion complex in the solution under this condition. It is worth mentioning that the stoichiometry of the inclusion complex in solution form by the titration was consistent with the results of the Job’s plot.

### 2.3. Electrochemical Study

Ni et al. reported that [Ni^II^(mnt)_2_]^2−^ functions as an electrochemical hydrogen production catalyst in aqueous or acetic acid/acetonitrile solutions with various acidity levels [17,18]. Thus, an electrochemical study was performed to compare the catalytic abilities of the free Ni complex **1** and inclusion complex **2**.

**Figure 6:**
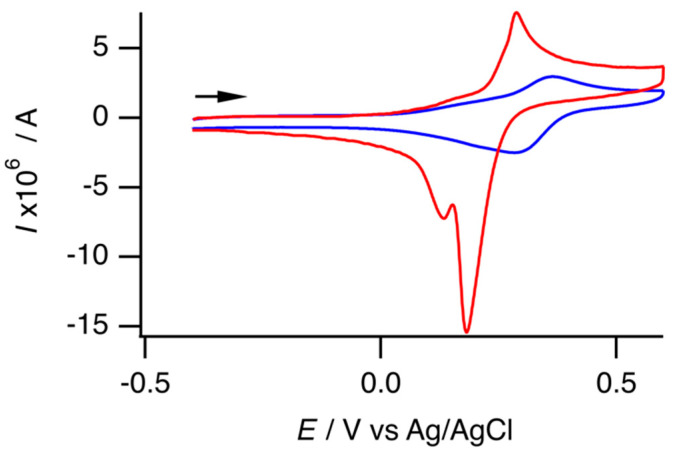
Cyclic voltammogram of the free Ni complex **1** (red) and inclusion complex **2** (blue) in 1 mol dm^−3^ Na_2_SO_4_/acetate buffer aqueous solution ([complex] = 5 × 10^−4^ mol dm^−3^; pH = 4.72). The arrow indicates the direction of the scan.

Cyclic voltammetry was performed to investigate the electrochemical properties of the free Ni complex and the inclusion complex in the aqueous solution of an acetate buffer (pH = 4.72). Figure 6 shows the cyclic voltammograms in the positive region. The free Ni complex had one oxidation peak (*E*_pa_ = 0.29 V vs. Ag/AgCl, *i*_pa_ = 6.82 × 10^−6^ A) and two reduction peaks (*E*_pc1_ = 0.18 V, *i*_pc1_ = 1.59 × 10^−5^ A; *E*_pc2_ = 0.13 V). Figure S18 shows the CVs at different scan rates (12–100 mV s^−1^). At lower scan rates, the voltammograms exhibited increased irreversibility. The CV at the slowest scan rate indicates a single irreversible redox process. In contrast, CVs at higher scan rates exhibited a quasi-reversible redox couple with an additional reduction peak. Table S1 summarizes the electrochemical data. Figures S19 and S20 show the plots of peak current versus scan rate and peak current versus the square root of the scan rate, respectively. The peak current of the more positive reduction peak appeared to correlate with the scan rate rather than with the square root of the scan rate. In addition, the current of the reduction peak was 2.3 times greater than that of the oxidation peak. Consequently, the free Ni complex exhibited a quasi-reversible redox couple, and adsorption on the working electrode occurred during the reduction process in the reverse scan. Furthermore, some oxidized species exhibited an additional reduction process, which was particularly noticeable at low scan rates. This irreversible behavior indicates that the free Ni complex could undergo an EC reaction. Oppositely, the inclusion complex exhibited one quasi-reversible redox couple (*E*_pa_ = 0.37 V, *i*_pa_ = 2.59 × 10^−6^ A; *E*_pc_ = 0.29 V, *i*_pc_ = 2.62 × 10^−6^ A). As shown in Figure S21, a low scan rate enhances the reversibility of the voltammogram; Table S2 summarizes the electrochemical data. Quantum calculations were conducted to identify the redox couple. Compared with the isolated state, the one-electron oxidized state lost an electron from a singly occupied molecular orbital (SOMO)(β). The SOMO(β) was similar to the highest-occupied molecular orbital of the isolated state, and both orbitals were largely attributed to the Ni atom rather than the mnt ligands (Figure S22). Thus, the redox couple was attributed to Ni(II)/Ni(III), and the result was consistent with that of a previous report [17]. The positive shift of the redox couple of the inclusion complex, compared with that of the free Ni complex, was an effect of encapsulation by β-CDs. The electrochemical reaction of the free Ni complex was probably caused by the structural change that occurred upon the oxidation of the Ni atom. Thus, the quasi-reversiblility of the inclusion complex suggested that the anion guest complex [Ni(mnt)_2_]^2−^ in the inclusion complex was too tightly enclosed to transform the structure upon oxidation. At all the scan rates, the anodic peak currents (*i*_pa_) of the inclusion complex were smaller than those of the free Ni complex. The decrease was consistent with a decrease in the diffusion constant due to encapsulation by CDs [23].

**Figure 7:**
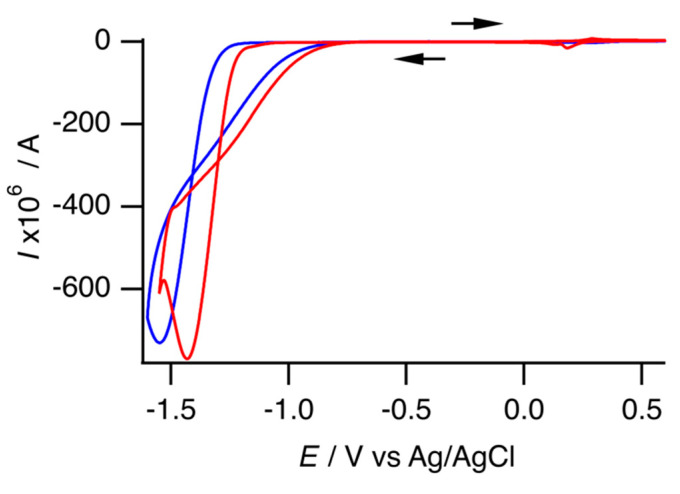
Cyclic voltammogram of free Ni complex **1** (red) and inclusion complex **2** (blue) under the same conditions as those depicted in Figure 6. The arrows indicate the direction of the scan.

Figure 7 shows a cyclic voltammogram with the negative region. The free Ni complex **1** exhibited one cathodic peak at −1.43 V, with a peak current that was 110 times that of the anodic peak current of the Ni(II)/Ni(III) redox couple. The prominent cathodic peak current was attributed to electrochemical hydrogen production, as previously mentioned by Ni et al. [17,18]. A CV was performed in DMF to examine the electrochemical behavior without a proton source (Figure S23, Table S3). Under this condition, the free Ni complex exhibited two reversible redox couples and one anodic peak. The reversible redox couple at −0.11 V vs. Ag/Ag^+^ would be for Ni(II)/Ni(III). Another reversible redox couple at −2.15 V vs. Ag/Ag^+^ would be for Ni(I)/Ni(II) (Figure S24). Assuming that |*E*_1/2_(Ni(II)/Ni(III)) − *E*_1/2_(Ni(I)/Ni(II))| was similar in both the aqueous and DMF solutions, an enhancement of the cathodic peak would occur with Ni(I)/Ni(II). Ni et al. speculated that the catalytic cycle would begin from the reduction of [Ni^II^(mnt)_2_]^2−^ [17].

With inclusion complex **2**, practically the same electrochemical behavior was observed; therefore, the inclusion complex also functioned as an electrochemical hydrogen production catalyst. Compared with the free Ni complex **1**, the redox potential of the Ni(II)/Ni(III) redox couple of the inclusion complex was a positive shift; however, the catalytic peak potential was a negative shift. This implies that the overpotential of the inclusion complex exceeded that of the free Ni complex. In addition, the result implies that encapsulation required more energy for electrochemical hydrogen production. Conversely, the catalytic peak currents of both the complexes were nearly identical. The redox behavior in the positive region indicates that the inclusion complex exhibited a smaller redox peak current, even at the same concentration as that of the free Ni complex. Therefore, the inclusion complex may have a better catalytic reaction rate than the free Ni complex. DuBois et al. reported that current enhancement *I*_cat_/*I*_p_ can be correlated with the turnover frequency *k*_cat_ of electrocatalysis, as expressed by the following Equation (3) [24]:

(3) $$\frac{I_{cat}}{I_{p}}=2.24\times n{\left(\frac{RTk_{cat}}{Fv}\right)}^{1/2}$$

where *I*_cat_ represents the catalytic peak current, *I*_p_ denotes the peak current in the absence of a substance, *n* is the number of electrons involved in the catalytic reduction, *R* represents the gas constant, *T* denotes the temperature, *F* is the Faraday constant, and *v* is the scan rate. This equation is applicable only under acid-independent conditions. Although it is unclear whether our experimental condition meets the acid-independent condition, we believe that the catalytic reaction rate can be assessed using *I*_cat_/*I*_p_. In this analysis, the anodic peak current of the Ni(II)/Ni(III) redox couple was used for *I*_p_ since measurements without protons were not feasible in this experiment. In addition, it was difficult to estimate the net catalytic peak current. One limitation was that the solvent exhibited a nonnegligible cathodic current, and another was that both complexes did not pass the “rinse test” (Figures S25–S27). Herein, we attempted to estimate the net catalytic peak current by subtracting the cathodic current of the solvent and the catalytic peak current of the “rinse test” from the observed catalytic peak current. Tables S1, S2 and S4 present all these data. Thus, the inclusion complex exhibits an *I*_cat_/*I*_p_ ratio that is 1.1 times greater than that of the free Ni complex. As illustrated in Figure S28, we also evaluated the catalytic reaction rate using foot-of-the-wave analysis [25,26]. However, since the half-wave potential and the peak current of the Ni(I)/Ni(II) redox couple are unknown, we used the *E*_pc_ of the catalytic reaction and the *i*_pa_ of the Ni(II)/Ni(III) redox couple instead. The turnover frequency *k*_cat_ was 4 × 10^7^ s^−1^ for the free Ni complex and 1 × 10^9^ s^−1^ for the inclusion complex. Although we did not consider these values to be quantitatively reliable, they did support our hypothesis that encapsulation enhances the catalytic reaction rate.

The encapsulation of the anion guest complex within the host molecules of β-CDs enhanced the catalytic reaction rate. The encapsulation placed the Ni ion as an active site in a hydrophilic location, which facilitated proton relay from the bulk area to the active site. This facilitation could have enhanced electrochemical hydrogen production [27,28,29]. The solvent in this study was aqueous; therefore, the enhancement might be more underestimated than that in an organic solvent. We attempted to compare the catalytic properties in a DMF solution (Figure S23 and Table S5). Under no-acid conditions, inclusion complex **2** exhibited almost the same electrochemical behavior as the free Ni complex **1**; thus, the inclusion complex would dissociate in DMF as an organic solvent and the estimation of catalytic behavior in an organic solvent is impossible.

## 3. Materials and Methods

### 3.1. General Methods

All the materials were purchased from commercial suppliers (Wako Pure Chemical Industries, Ltd. (Osaka, Japan); Kanto Chemical Co., Inc. (Tokyo, Japan); Tokyo Chemical Industry Co., Ltd. (Tokyo, Japan); Euriso-Top Ltd. (St.-Aubin, France); and Sigma-Aldrich Co., Ltd. (Darmstadt, Germany) and were used without further purification.

A bulk sample of the inclusion complex was used for all the measurements except for the single-crystal X-ray investigation, for which air-dried precipitates were used. The single-crystal study revealed that the inclusion complex contained 20 water molecules. Elemental analysis revealed a water content of 20 molecules for the bulk samples. Powder X-ray diffraction (PXRD) revealed that the structure of the bulk sample was almost the same as that of the single crystal (Figure S29).

C, H, and N analysis was conducted by the Service Center of the Elementary Analysis of Organic Compounds of Kyushu University. The infrared (IR) spectrum was recorded using a JASCO FT/IR 6100 Fourier transform IR spectrometer (JASCO Corp., Tokyo, Japan) with KBr pellets. The proton nuclear magnetic resonance (^1^H NMR) (400 MHz) spectrum was recorded in D_2_O, using a Bruker Avance III HD spectrometer (Bruker Corp., Billerica, MA, USA) with 3-(trimethylsilyl)-1-propanesulfonic acid sodium salt (DSS) as an internal standard (*δ*_H_ of (C*H*_3_)_3_Si− = 0.00). The concentrations of inclusion complex **2** and β-CD were 1 × 10^−3^ and 2 × 10^−3^ mol dm^−3^, respectively. The ^13^C NMR (101 MHz) spectrum was also recorded under almost the same conditions as ^1^H NMR. DDS was used as an internal standard (*δ*_C_ of (*C*H_3_)_3_Si− = 0.00). The concentrations of inclusion complex **2** and β-CD were 1 × 10^−3^ and moderately thick, respectively. The absorption spectrum was recorded using a SHIMADZU UV-3600 ultraviolet-visible near-infrared (UV–VIS–NIR) spectrophotometer (Shimadzu Corp., Kyoto, Japan). The optical path length was 1 mm. Curve fitting was performed to estimate the association constants, using SPANA (ver. 5.5.79) [30]. Cyclic voltammetry (CV) was performed using a BAS BAS100B/W electrochemical workstation (Bioanalytical Systems, Inc., West Lafayette, IN, USA). A three-electrode cell with a glassy carbon electrode was used as the working electrode, and a Pt coil electrode was used as the counter electrode. Finally, an Ag/AgCl electrode (for an aqueous solution) or a homemade Ag/Ag^+^ electrode (for an organic solvent) was used as the reference electrode. The *E*_1/2_ of a [Fe(CN)_6_]^4−/3−^ couple, acting as an external reference, was 0.29 V vs. Ag/AgCl. The *E*_1/2_ of a ferrocene/ferrocenium couple, used as an external reference, was 0.05 V vs. Ag/Ag^+^. The 0.1 mol dm^−3^ Na_2_SO_4_/acetate aqueous buffer solution was used as an aqueous electrolyte, and the concentration of the complex was 5 × 10^−4^ mol dm^−3^. The acetate aqueous buffer solution was prepared by mixing 100 mL of 0.1 mol dm^−3^ acetic acid solution with 100 mL of 0.1 mol dm^−3^ sodium acetate solution. The pH of the buffer solution was 4.72. In addition, the organic medium of a 0.1 mol dm^−3^ tetrabutylammonium hexafluorophosphate/*N,N*-dimethylformamide (DMF) solution was used for an organic electrolyte, and the concentration of the complex was 1 × 10^−3^ mol dm^−3^. The scan rate was 100 mV s^−1^, except when otherwise noted. All electrochemical measurements were conducted under a nitrogen atmosphere. The pH was measured using a DKK-TOA HM-40S pH meter (DKK-TOA Corp., Tokyo, Japan) with a two-point calibration setup (pH levels of 4.01 and 6.89). The PXRD data were recorded using a Rigaku MultiFlex X-ray diffractometer (Rigaku Corp., Tokyo, Japan) with Cu Kα radiation at a wavelength of 1.542 Å. Electrospray ionization mass (ESI-mass) spectral data were obtained using a JEOL JMS-T100CS spectrometer (JEOL Ltd., Tokyo, Japan). All the measurements were performed at room temperature.

### 3.2. Synthesis

K_2_[Ni(mnt)_2_] (**1**) was prepared using a similar, previously reported method [31]. Elemental analysis (%) found: C, 22.2; H, 0.1; N, 12.8. Calc. for C_8_K_2_N_4_Ni_1_S_4_·0.5H_2_O: C, 22.5; H, 0.2; N, 13.1.

K_2_{[Ni(mnt)_2_]@(β-CD)_2_} (**2**)**.** NiSO_4_·6H_2_O (49.9 mg, 0.190 mmol), Na_2_mnt (70.0 mg, 0.376 mmol), and β-CD (442.5 mg, 0.390 mmol) were dissolved in H_2_O (15 mL). The resulting transparent red-brown solution was then evaporated to concentrate up to ca. 6 mL using a rotary evaporator. Excess CH_3_COOK was added to the solution, and a red microcrystalline product was obtained via a vapor diffusion method (adding methanol vapor into the solution). The crude product was dissolved in a minimal amount of water, and excess ethanol was added to the solution. The resulting red precipitate was filtered. The purification was conducted thrice. Yield **2**·20H_2_O (332.2 mg, 57.4%). Elemental analysis (%) Found: C, 36.0; H, 6.0; N, 1.4. Calc. for C_92_H_140_K_2_N_4_NiO_70_S_4_·20H_2_O: C, 36.3; H, 6.0; N, 1.8. FT-IR *ν*_max_/cm^−1^ 2198 (CN). ESI-Mass *m*/*z* 1304 (1304, (M-2K)^2−^). ^1^H NMR (400 MHz, D_2_O; DSS) 5.00 (14 H, d, *J* = 3.6 Hz), 3.83–3.96 (56 H, m), 3.54–3.59 (28 H, m). ^13^C NMR (101 MHz, D_2_O; DSS) 104.15, 82.82, 75.20, 74.30, 73.57, 62.31.

### 3.3. X-Ray Crystallography

Single crystals of **2**, suitable for single-crystal X-ray analysis, were obtained by vapor diffusion (adding methanol vapor into an H_2_O solution of **2**) at room temperature. The data were collected using a RIGAKU R-AXIS RAPID II IP diffractometer. A multiscan absorption correction was applied to the intensity data. The structure was solved using a direct method (SHELXT-2018/2) [32] and refined via the full-matrix least-squares method on *F*^2^ (SHELXL-2018/3) [32], using the Yadokari-XG software package (Rev. 1078) [33]. All nonhydrogen atoms were refined using anisotropic parameters. The H atoms of water were not included in the final refinement. Other H atoms were included in the calculated positions and then refined using a riding model.

Crystal data for **2**·20H_2_O: C_92_H_180_K_2_N_4_NiO_90_S_4_, *M* = 3047.54 g mol^−3^, *T* = 100 (2) K, monoclinic, *C*2, *a* = 18.8845 (4) Å, *b* = 24.6655 (4) Å, *c* = 15.6064 (3) Å, *β* = 108.542 (8)°, *V* = 6892.0 (4) Å^3^, *Z* = 2, *D*_calc_ = 1.469 g cm^−3^, *μ* = 0.376 mm^−1^, crystal size 0.56 × 0.26 × 0.15 mm^3^, reflections collected 45,106, independent reflections 12,549, *R*_int_ = 0.0264, goodness-of-fit on *F*^2^ = 1.110, *R*_1_ = 0.0754, w*R*_2_ = 0.1970 (for *I* > 2*σ* (*I*)), *R*_1_ = 0.0767, w*R*_2_ = 0.1992 (for all data) (*R*_1_ = ∑||*F*_o_| − |*F*_c_||/∑|*F*_o_|. w*R*_2_ = [∑w(*F*_o_^2^ − *F*_c_^2^)^2^/∑w(*F*_o_^2^)^2^]^1/2^), absolute structure parameter = 0.045 (3). CCDC 2,377,459 contains the supplementary crystallographic data for this paper. The data can be obtained free of charge via https://www.ccdc.cam.ac.uk/structures/ (or from the CCDC, 12 Union Road, Cambridge CB2 1EZ, UK; Fax: +44-1223-336033; e-mail: deposit@ccdc.cam.ac.uk).

### 3.4. Computational Details

The Gaussian 09 (Rev. D.01) program was used for all the calculations [34]. To estimate the oxidized and reduced states of [Ni(mnt)_2_]^2−^, density functional theory (DFT) calculations were performed. The B3LYP functional was used, and the basis set SDD and 6-31G* were selected for Ni and other atoms, respectively. The initial geometry for geometry optimization was the modified X-ray structure of the inclusion complex. Optimization for the isolated state was performed using the modeled structure; the geometry obtained was defined as “square-planar geometry”. Thereafter, the as-optimized structure was used as the initial structure for the second optimization for the oxidized and reduced states. The solvent effect of water was applied using the default Gaussian polarizable continuum model implementation. Facio (ver. 23.1.5) was used for molecular modeling [35], and Gabedit (ver. 2.5.1) was used to visualize the molecular orbitals [36]. The optimized structure of the inclusion complex was also obtained with the aforementioned method.

The simulation of the IR spectrum was performed using a vibrational frequency calculation with an optimized structure. For simplicity, the solvent effect of water was applied without a scaling factor. Facio (ver. 23.1.5) was used to visualize the calculated IR spectrum.

To simulate the absorption spectrum, time-dependent DFT computation was conducted to calculate the excited states with the optimized structure, using the aforementioned method. In the case of the calculation of [Ni(mnt)_2_]^2−^ with the included geometry in β-CDs, the structure was extracted from the optimized inclusion complex and was used without further optimization. The geometry was defined as “slightly distorted square-planar geometry”. The solvent effect of water or heptane was applied. GaussSum (ver. 3.0) was used to visualize the simulated spectrum [37].

Selected bond distances and angles of the isolated state geometries are listed in Table S6. Those from the single-crystal X-ray study are also included in Table S6. The table suggests that the calculated structures reproduce the original structures.

The weak interaction between β-CDs and cyano groups of the anion guest complex in the inclusion complex was analyzed using multifunctional wave function analysis software (Multiwfn) (Ver. 3.8) [38] and a visual molecular dynamics (VMD) package (Ver. 1.9.3) [39], with an independent gradient model based on the Hirshfeld partition of molecular density (IGMH) method.

## 4. Conclusions

For this article, we synthesized an inclusion complex K_2_{[Ni(mnt)_2_]@(β-CD)_2_} and determined its structure via X-ray crystallography. The inclusion complex was constructed using [Ni(mnt)_2_]^2−^ as an anion guest complex and two β-CDs as a host supramolecule; the structure of the complex remained the same, even in an aqueous solution state. The association constants were estimated at 1.1(7) × 10^4^ and 1.8(5) × 10^4^ mol^−1^ dm^3^ for *K*_1_ and *K*_2_, respectively. The catalytic behavior of the inclusion complex and the free Ni complex K_2_{[Ni(mnt)_2_] for electrochemical hydrogen production in an aqueous solution was investigated, and the results showed that the inclusion complex was observed to have a better catalytic reaction rate than the free Ni complex. This enhancement resulted from the encapsulation of the guest anion complex in the host supramolecule.

However, a detailed analysis of the enhancement proved difficult, due to a failure on a rinse test. Moreover, the encapsulation also showed a negative impact on potential. Currently, we are attempting to research another inclusion complex that conquers these problems to demonstrate that the encapsulation of guest molecules within host structures can enhance their catalytic behavior.

## Data Availability

The data supporting this article have been included within the article and Supplementary Materials. Further inquiries can be directed to the corresponding author.

## Supplementary Materials

The following supporting information can be downloaded at https://www.mdpi.com/article/10.3390/molecules29245858/s1: Figure S1: Single X-ray crystallography; Figure S2: Numbering scheme; Figure S3: Single X-ray crystallography; Figure S4: Interaction analysis by the IGMH method; Figure S5: IR spectra; Figure S6: Simulated IR spectra; Figures S7 and S8: ^1^H NMR spectra; Figure S9: Histograms of Ni–S bond lengths in [Ni(mnt)_2_]^n−^; CDS refcodes of [Ni^II^(mnt)_2_]^2−^ for Figure S9; CDS refcodes of [Ni^III^(mnt)_2_]^−^ for Figure S9; Figures S10 and S11: ^13^C NMR spectra; Figures S12–S14: UV–VIS spectra; Figures S15–S17: Simulated UV–Vis spectra; Figure S18: Cyclic voltammograms; Figure S19: Plot of peak current versus scan rate; Figure S20: Plot of peak current versus square root of scan rate; Figure S21: Cyclic voltammograms; Figure S22: Molecular orbitals; Figure S23: Cyclic voltammograms in an organic solvent; Figure S24: Molecular orbitals; Figure S25: Cyclic voltammograms; Figures S26 and S27: Catalytic behavior; Figure S28: Foot-of-the-wave analysis; Figure S29: Powder X-ray diffraction patterns; Figure S30: Electrospray ionization mass spectra; Parameters of the curve-fitting simulation performed for the association constants (inset of Figure 5); Tables S1–S3: Electrochemical data; Table S4: Catalytic data; Table S5: Electrochemical data; Table S6: Selected bond distances, angles and a dihedral angle; Tables S7–S10: Coordinates of optimized structures. CIF data of complex **2** and the evaluation at the checkCIF website can also be obtained from the same website.
