# Supplementary material for: Structure of Ni(II) Inclusion Complex in Solid/Solution States and the Enhancement of Catalytic Behavior in Electrochemical Hydrogen Production
Source: Molecules. 2024 Dec 12;29(24):5858. doi: 10.3390/molecules29245858 (PMC11678872; doi:10.3390/molecules29245858)

checkCIF/PLATON report

Structure factors have been supplied for datablock(s) Nimnt2bCD

THIS REPORT IS FOR GUIDANCE ONLY. IF USED AS PART OF A REVIEW PROCEDURE FOR PUBLICATION, IT SHOULD NOT REPLACE THE EXPERTISE OF AN EXPERIENCED CRYSTALLOGRAPHIC REFEREE.

No syntax errors found. CIF dictionary Interpreting this report

Datablock: Nimnt2bCD

Bond precision:	C-C = 0.0093 A		Wavelength=0.71075
Cell:	a=18.8845(4)	b=24.6655(4)	c=15.6064(3)
	alpha=90	beta=108.542(8)	gamma=90
Temperature:	100 K		

	Calculated	Reported
Volume	6892.1(4)	6892.0(4)
Space group	C 2	C 2
Hall group	C 2y	C 2y
Moiety formula	2(C42 H70 K O37), C8 N4 Ni S4, 16(O)	?
Sum formula	C92 H140 K2 N4 Ni O90 S4	C92 H180 K2 N4 Ni O90 S4
Mr	3007.22	3047.54
Dx,g cm-3	1.449	1.469
Z	2	2
Mu (mm-1)	0.376	0.376
F000	3140.0	3220.0
F000'	3144.71	
h,k,lmax	22,29,18	22,29,18
Nref	12624[6471]	12549
Tmin,Tmax	0.889,0.945	0.775,1.000
Tmin'	0.810	

Correction method= # Reported T Limits: Tmin=0.775 Tmax=1.000
AbsCorr = MULTI-SCAN

Data completeness= 1.94/0.99 Theta(max)= 25.345

R(reflections)= 0.0754(12141) wR2(reflections)= 0.1992(12549)

S = 1.110 Npar= 920

The following ALERTS were generated. Each ALERT has the format
test-name_ALERT_alert-type_alert-level.
Click on the hyperlinks for more details of the test.

Alert level B

PLAT043_ALERT_1_B Calculated and Reported Mol. Weight Differ by .. 40.32 Check
PLAT241_ALERT_2_B High 'MainMol' Ueq as Compared to Neighbors of S1 Check

Author Response: The alert would be caused from loose inclusion between guest and host molecules. However, this does not indicate an incorrect atom-type assignment.

PLAT250_ALERT_2_B Large U3/U1 Ratio for <U(i,j)> Tensor(Resd 2) 5.4 Note

Author Response: The large U3/U1 ratio is attributed to the badly disordered water molecules.

PLAT306_ALERT_2_B Isolated Oxygen Atom (H-atoms Missing ?) .... O37 Check

Author Response: Hydrogen atoms of water molecules are not observed in residual electron density maps, and therefore are not included in the structure model.

PLAT306_ALERT_2_B Isolated Oxygen Atom (H-atoms Missing ?) .... O38 Check

Author Response: Hydrogen atoms of water molecules are not observed in residual electron density maps, and therefore are not included in the structure model.

PLAT306_ALERT_2_B Isolated Oxygen Atom (H-atoms Missing ?) .... O39 Check

Author Response: Hydrogen atoms of water molecules are not observed in residual electron density maps, and therefore are not included in the structure model.

PLAT306_ALERT_2_B Isolated Oxygen Atom (H-atoms Missing ?) .... O40 Check

Author Response: Hydrogen atoms of water molecules are not observed in residual electron density maps, and therefore are not included in the structure model.

```
PLAT306_ALERT_2_B Isolated Oxygen Atom (H-atoms Missing ?) .......        O41 Check
```

Author Response: Hydrogen atoms of water molecules are not observed in residual electron density maps, and therefore are not included in the structure model.

```
PLAT306_ALERT_2_B Isolated Oxygen Atom (H-atoms Missing ?) .......        O42 Check
```

Author Response: Hydrogen atoms of water molecules are not observed in residual electron density maps, and therefore are not included in the structure model.

```
PLAT306_ALERT_2_B Isolated Oxygen Atom (H-atoms Missing ?) .......        O43 Check
```

Author Response: Hydrogen atoms of water molecules are not observed in residual electron density maps, and therefore are not included in the structure model.

```
PLAT306_ALERT_2_B Isolated Oxygen Atom (H-atoms Missing ?) .......        O45 Check
```

Author Response: Hydrogen atoms of water molecules are not observed in residual electron density maps, and therefore are not included in the structure model.

```
PLAT420_ALERT_2_B D-H Bond Without Acceptor  O11       --H11B     .     Please Check
PLAT420_ALERT_2_B D-H Bond Without Acceptor  O14       --H14A     .     Please Check
PLAT420_ALERT_2_B D-H Bond Without Acceptor  O21       --H21A     .     Please Check
PLAT430_ALERT_2_B Short Inter D...A Contact  O36       ..O45      .       2.73 Ang.
                                                       x,y,z  =      1_555 Check
```

Author Response: The atoms represent water molecules. Their hydrogen atoms are not observed in residual electron density maps, and therefore are not included in the structure model.

```
PLAT430_ALERT_2_B Short Inter D...A Contact  O36       ..O42      .       2.76 Ang.
                                                   2-x,y,1-z  =      2_756 Check
```

Author Response: The atoms represent water molecules. Their hydrogen atoms are not observed in residual electron density maps, and therefore are not included in the structure model.

```
PLAT430_ALERT_2_B Short Inter D...A Contact  O37       ..O37      .       2.74 Ang.
                                                   1-x,y,1-z  =      2_656 Check
```

Author Response: The atoms represent water molecules. Their hydrogen atoms are not observed in residual electron density maps, and therefore are not included in the structure model.

```
PLAT430_ALERT_2_B Short Inter D...A Contact  O38       ..N2       .       2.82 Ang.
                                                    x,y,z  =      1_555 Check
```

Author Response: The atoms represent water molecules. Their hydrogen atoms are not observed in residual electron density maps, and therefore are not included in the structure model.

```
PLAT430_ALERT_2_B Short Inter D...A Contact  O38       ..N2       .       2.82 Ang.
                                                1-x,y,1-z  =      2_656 Check
```

Author Response: The atoms represent water molecules. Their hydrogen atoms are not observed in residual electron density maps, and therefore are not included in the structure model.

```
PLAT430_ALERT_2_B Short Inter D...A Contact  O39       ..O42      .       2.72 Ang.
                                        -1/2+x,1/2+y,z  =      3_455 Check
```

Author Response: The atoms represent water molecules. Their hydrogen atoms are not observed in residual electron density maps, and therefore are not included in the structure model.

```
PLAT430_ALERT_2_B Short Inter D...A Contact  O39       ..O41      .       2.78 Ang.
                                         1/2+x,1/2+y,z  =      3_555 Check
```

Author Response: The atoms represent water molecules. Their hydrogen atoms are not observed in residual electron density maps, and therefore are not included in the structure model.

```
PLAT430_ALERT_2_B Short Inter D...A Contact  O43       ..O44      .       2.71 Ang.
                                                    x,y,z  =      1_555 Check
```

Author Response: The atoms represent water molecules. Their hydrogen atoms are not observed in residual electron density maps, and therefore are not included in the structure model.

```
PLAT910_ALERT_3_B Missing # of FCF Reflection(s) Below Theta(Min).         15 Note
             2  0  0,   1  1  0,   0  2  0,   2  2  0,   1  3  0,  -2  0  1,
             0  0  1,  -1  1  1,   1  1  1,  -2  2  1,   0  2  1,  -1  3  1,
            -2  0  2,   0  0  2,  -1  1  2,
```

Author Response: The unit cell is reasonable large and these low angle reflections are probably missing due to the beamstop.

```
PLAT934_ALERT_3_B Number of (Iobs-Icalc)/Sigma(W) > 10 Outliers ..          2 Check
                -4  0  2,   0  0  4,
```

Author Response: Water molecules make the crystal quality low, that could cause the problem.

```
PLAT987_ALERT_1_B The Flack x is >> 0 -  Do a BASF/TWIN Refinement     Please Check
```

Author Response: The Flack parameter is near to zero [0.045(3)]. And beta-cyclodextrin is chiral molecule.

Alert level C

```
PLAT041_ALERT_1_C Calc. and Reported SumFormula    Strings  Differ     Please Check
              Calc: C92 H140 K2 N4 Ni O90 S4
              Rep.: C92 H180 K2 N4 Ni O90 S4
PLAT068_ALERT_1_C Reported F000 Differs from Calcd (or Missing)...     Please Check
PLAT090_ALERT_3_C Poor Data / Parameter Ratio (Zmax > 18) ........       7.01 Note
PLAT094_ALERT_2_C Ratio of Maximum / Minimum Residual Density ....       2.96 Report
PLAT220_ALERT_2_C NonSolvent   Resd 1  O   Ueq(max)/Ueq(min) Range        4.3 Ratio
PLAT222_ALERT_3_C NonSolvent Resd 1  H   Uiso(max)/Uiso(min) Range        5.7 Ratio
PLAT242_ALERT_2_C Low    'MainMol' Ueq as Compared to Neighbors of        C17 Check
PLAT242_ALERT_2_C Low    'MainMol' Ueq as Compared to Neighbors of        Ni1 Check
PLAT242_ALERT_2_C Low    'MainMol' Ueq as Compared to Neighbors of         C1 Check
PLAT260_ALERT_2_C Large Average Ueq of Residue Including       O37      0.263 Check
PLAT260_ALERT_2_C Large Average Ueq of Residue Including       O38      0.221 Check
PLAT260_ALERT_2_C Large Average Ueq of Residue Including       O40      0.114 Check
PLAT260_ALERT_2_C Large Average Ueq of Residue Including       O43      0.122 Check
PLAT260_ALERT_2_C Large Average Ueq of Residue Including       O45      0.151 Check
PLAT260_ALERT_2_C Large Average Ueq of Residue Including       O46      0.225 Check
PLAT341_ALERT_3_C Low Bond Precision on  C-C Bonds ...............    0.00926 Ang.
PLAT918_ALERT_3_C Reflection(s) with I(obs) much Smaller I(calc) .          2 Check
PLAT973_ALERT_2_C Check Calcd Positive Resid. Density on        K2       1.48 eA-3
PLAT975_ALERT_2_C Check Calcd Resid. Dens.  0.94Ang From O46     .       0.66 eA-3
PLAT975_ALERT_2_C Check Calcd Resid. Dens.  0.90Ang From O43     .       0.52 eA-3
PLAT975_ALERT_2_C Check Calcd Resid. Dens.  1.05Ang From O45     .       0.49 eA-3
PLAT975_ALERT_2_C Check Calcd Resid. Dens.  0.67Ang From O38     .       0.49 eA-3
PLAT976_ALERT_2_C Check Calcd Resid. Dens.  1.05Ang From O14     .      -0.48 eA-3
PLAT977_ALERT_2_C Check Negative Difference Density on H14A      .      -0.48 eA-3
```

Alert level G

```
FORMU01_ALERT_2_G  There is a discrepancy between the atom counts in the
            _chemical_formula_sum and the formula from the _atom_site* data.
            Atom count from _chemical_formula_sum:C92 H180 K2 N4 Ni1 O90 S4
            Atom count from the _atom_site data:  C92 H140 K2 N4 Ni1 O90 S4
CELLZ01_ALERT_1_G Difference between formula and atom_site contents detected.
CELLZ01_ALERT_1_G WARNING: H atoms missing from atom site list. Is this intentional?
```

```
From the CIF: _cell_formula_units_Z    2
From the CIF: _chemical_formula_sum  C92 H180 K2 N4 Ni O90 S4
TEST: Compare cell contents of formula and atom_site data

atom    Z*formula  cif sites diff
C        184.00    184.00    0.00
H        360.00    280.00   80.00
K          4.00      4.00    0.00
N          8.00      8.00    0.00
Ni         2.00      2.00    0.00
O        180.00    180.00    0.00
S          8.00      8.00    0.00
```

```
PLAT004_ALERT_5_G Polymeric Structure Found with Maximum Dimension          3 Info
PLAT007_ALERT_5_G Number of Unrefined Donor-H Atoms .............          21 Report
           H1    H3    H4    H6A   H8A   H9A   H11B  H13A  H14A  H16A  H18A
           H19A  H21A  H23B  H24A  H26A  H28A  H29B  H31A  H33A  H34A
PLAT033_ALERT_4_G Flack x Value Deviates > 3.0 * sigma from Zero .      0.045 Note
PLAT083_ALERT_2_G SHELXL Second Parameter in WGHT  Unusually Large      10.28 Why ?
PLAT299_ALERT_4_G Atom Site Occupancy Constrained at ............         0.5 Check
           K1      K2      O44     O48
PLAT301_ALERT_3_G Main Residue  Disorder .............(Resd    1)          3% Note
PLAT302_ALERT_4_G Anion/Solvent/Minor-Residue Disorder (Resd   11)       100% Note
PLAT302_ALERT_4_G Anion/Solvent/Minor-Residue Disorder (Resd   12)       100% Note
PLAT303_ALERT_2_G Full Occupancy Atom H1        with # Connections       1.50 Check
PLAT303_ALERT_2_G Full Occupancy Atom H11B      with # Connections       1.50 Check
PLAT303_ALERT_2_G Full Occupancy Atom H21A      with # Connections       1.50 Check
PLAT311_ALERT_2_G Isolated Disordered Oxygen Atom (No H's ?) .....        O44 Check
PLAT311_ALERT_2_G Isolated Disordered Oxygen Atom (No H's ?) .....        O46 Check
PLAT311_ALERT_2_G Isolated Disordered Oxygen Atom (No H's ?) .....        O47 Check
PLAT480_ALERT_4_G Long H...A H-Bond Reported H11A     ..O12       .       2.65 Ang.
PLAT480_ALERT_4_G Long H...A H-Bond Reported H12      ..S1        .       2.93 Ang.
PLAT480_ALERT_4_G Long H...A H-Bond Reported H13      ..O28       .       2.62 Ang.
PLAT480_ALERT_4_G Long H...A H-Bond Reported H9A      ..O33       .       2.63 Ang.
PLAT480_ALERT_4_G Long H...A H-Bond Reported H17A     ..O17       .       2.66 Ang.
PLAT480_ALERT_4_G Long H...A H-Bond Reported H19      ..O36       .       2.65 Ang.
PLAT480_ALERT_4_G Long H...A H-Bond Reported H24      ..S2        .       2.90 Ang.
PLAT480_ALERT_4_G Long H...A H-Bond Reported H24A     ..O18       .       2.64 Ang.
PLAT480_ALERT_4_G Long H...A H-Bond Reported H34A     ..O8        .       2.64 Ang.
PLAT780_ALERT_1_G Coordinates do not Form a Properly Connected Set     Please Do !
PLAT794_ALERT_5_G Tentative Bond Valency for Ni1       (III)      .       2.82 Info
PLAT860_ALERT_3_G Number of Least-Squares Restraints ............          22 Note
PLAT899_ALERT_4_G SHELXL2018 is Outdated   and Succeeded by SHELXL     2019/3 Note
PLAT912_ALERT_4_G Missing # of FCF Reflections Above STh/L=  0.600          4 Note
PLAT969_ALERT_5_G The 'Henn et al.' R-Factor-gap value ..........      7.806 Note
           Predicted wR2: Based on SigI**2  2.55 or SHELX Weight 17.95
PLAT978_ALERT_2_G Number C-C Bonds with Positive Residual Density.          0 Info
```

---

 0 **ALERT level A** = Most likely a serious problem - resolve or explain
25 **ALERT level B** = A potentially serious problem, consider carefully
24 **ALERT level C** = Check. Ensure it is not caused by an omission or oversight
33 **ALERT level G** = General information/check it is not something unexpected

7 ALERT type 1 CIF construction/syntax error, inconsistent or missing data

```
48 ALERT type 2 Indicator that the structure model may be wrong or deficient
 8 ALERT type 3 Indicator that the structure quality may be low
15 ALERT type 4 Improvement, methodology, query or suggestion
 4 ALERT type 5 Informative message, check
```

It is advisable to attempt to resolve as many as possible of the alerts in all categories. Often the minor alerts point to easily fixed oversights, errors and omissions in your CIF or refinement strategy, so attention to these fine details can be worthwhile. In order to resolve some of the more serious problems it may be necessary to carry out additional measurements or structure refinements. However, the purpose of your study may justify the reported deviations and the more serious of these should normally be commented upon in the discussion or experimental section of a paper or in the "special_details" fields of the CIF. checkCIF was carefully designed to identify outliers and unusual parameters, but every test has its limitations and alerts that are not important in a particular case may appear. Conversely, the absence of alerts does not guarantee there are no aspects of the results needing attention. It is up to the individual to critically assess their own results and, if necessary, seek expert advice.

Publication of your CIF in IUCr journals

A basic structural check has been run on your CIF. These basic checks will be run on all CIFs submitted for publication in IUCr journals (*Acta Crystallographica*, *Journal of Applied Crystallography*, *Journal of Synchrotron Radiation*); however, if you intend to submit to *Acta Crystallographica Section C* or *E* or *IUCrData*, you should make sure that full publication checks are run on the final version of your CIF prior to submission.

Publication of your CIF in other journals

Please refer to the *Notes for Authors* of the relevant journal for any special instructions relating to CIF submission.

PLATON version of 22/08/2024; check.def file version of 21/08/2024

Datablock Nimnt2bCD - ellipsoid plot

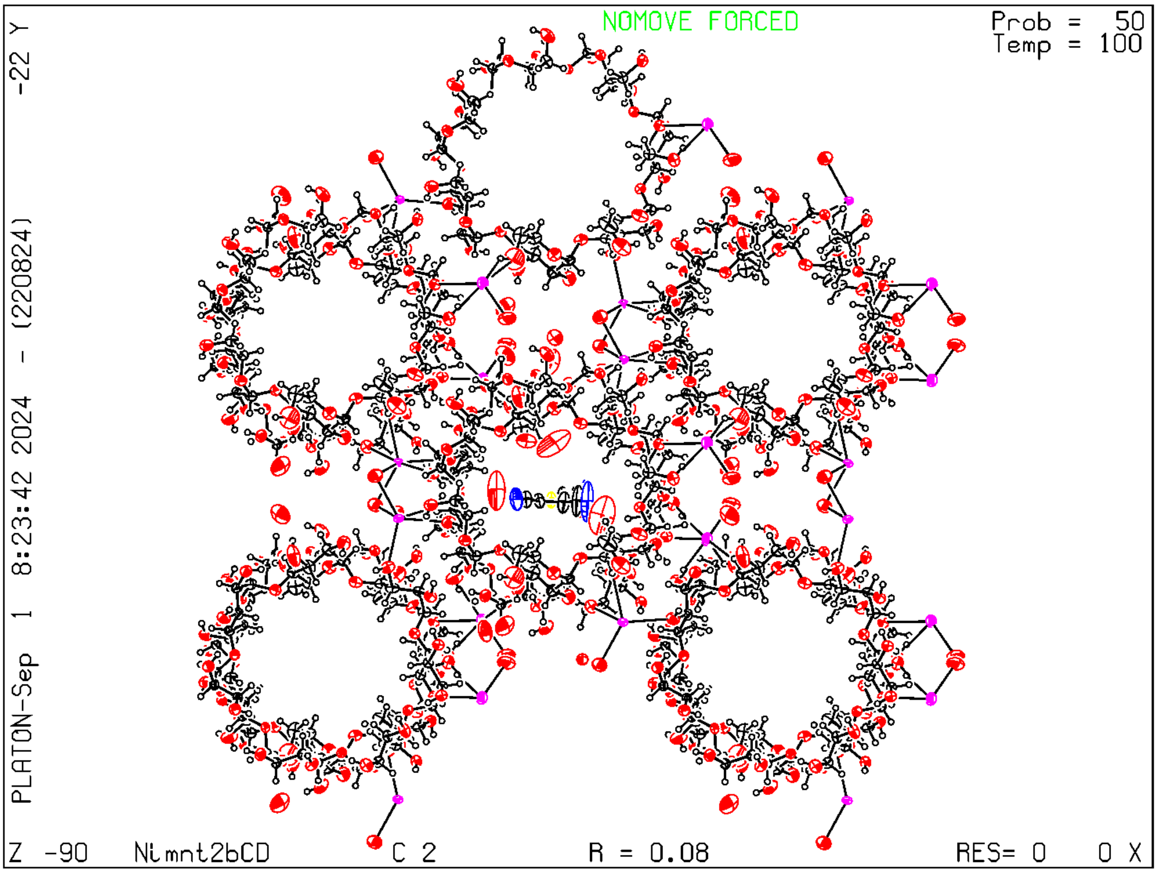

Supplement: Supplementary file 1 [file molecules-29-05858-s001.zip › checkcif.pdf]
