# Supplementary material for: Structure of Ni(II) Inclusion Complex in Solid/Solution States and the Enhancement of Catalytic Behavior in Electrochemical Hydrogen Production
Source: Molecules. 2024 Dec 12;29(24):5858. doi: 10.3390/molecules29245858 (PMC11678872; doi:10.3390/molecules29245858)
Supplement: Supplementary file 1 [file molecules-29-05858-s001.zip › sm.pdf]

Supplementary Materials

Structure of Ni(II) Inclusion Complex in Solid/Solution States and the Enhancement of Catalytic Behavior in Electrochemical Hydrogen Production

Table of contents

Figure S1	Single X-ray crystallography of the inclusion complex **2**
Figure S2	Numbering scheme for the $[Ni(mnt)_2]^{2-}$
Figure S3	Single X-ray crystallography of the inclusion complex **2**
Figure S4	Interaction analysis between $[Ni(mnt)_2]^{2-}$ and β-CDs by the IGMH method
Figure S5	IR spectrum of the free Ni complex **1** and inclusion complex **2**
Figure S6	Simulated IR spectra
Figures S7–8	^{1}H NMR spectra of the inclusion complex **2** and β-CD
Figure S9	Histograms of Ni–S bond lengths in $[Ni(mnt)_2]^{n-}$

CDS refcodes of $[Ni^{II}(mnt)_2]^{2-}$ for Figure S9

CDS refcodes of $[Ni^{III}(mnt)_2]^{2-}$ for Figure S9

Figures S10–S11	^{13}C NMR spectra of the inclusion complex **2** and β-CD
Figures S12–S14	UV–VIS spectra of the free Ni complex **1** with various amounts of β -CD
Figures S15–S17	Simulated UV–Vis spectra
Figure S18	Cyclic voltammograms of the free Ni complex **1** at various scan rates
Figure S19	Plot of peak current versus scan rate for the free Ni complex **1**

Figure S20 Plot of peak current versus square root of scan rate for the free Ni complex **1**

Figure S21 Cyclic voltammograms of the inclusion complex **2** at various scan rates

Figure S22 Molecular orbitals

Figure S23 Cyclic voltammograms of the free Ni complex **1** and inclusion complex **2** in an organic solvent

Figure S24 Molecular orbitals

Figure S25 Cyclic voltammograms of the baseline, free Ni complex **1**, and inclusion complex **2**

Figures S26–S27 Catalytic behavior

Figure S28 Foot-of-the-wave analysis

Figure S29 Powder X-ray diffraction patterns of the inclusion complex **2**

Figure S30 Electrospray ionization mass spectra

Parameters of the curve-fitting simulation performed for the association constants (inset of Figure 5)

Table S1 Electrochemical data of the free Ni complex **1** at various scan rates

Table S2 Electrochemical data of the inclusion complex **2** at various scan rates

Table S3 Electrochemical data of the free Ni complex **1** in an organic solvent

Table S4 Catalytic data

Table S5 Electrochemical data of the inclusion complex **2** in an organic solvent

Table S6 Selected bond distances, angles and a dihedral angle of $[Ni(mnt)_2]^{2-}$

Tables S7–S10 Coordinates of optimized structures.

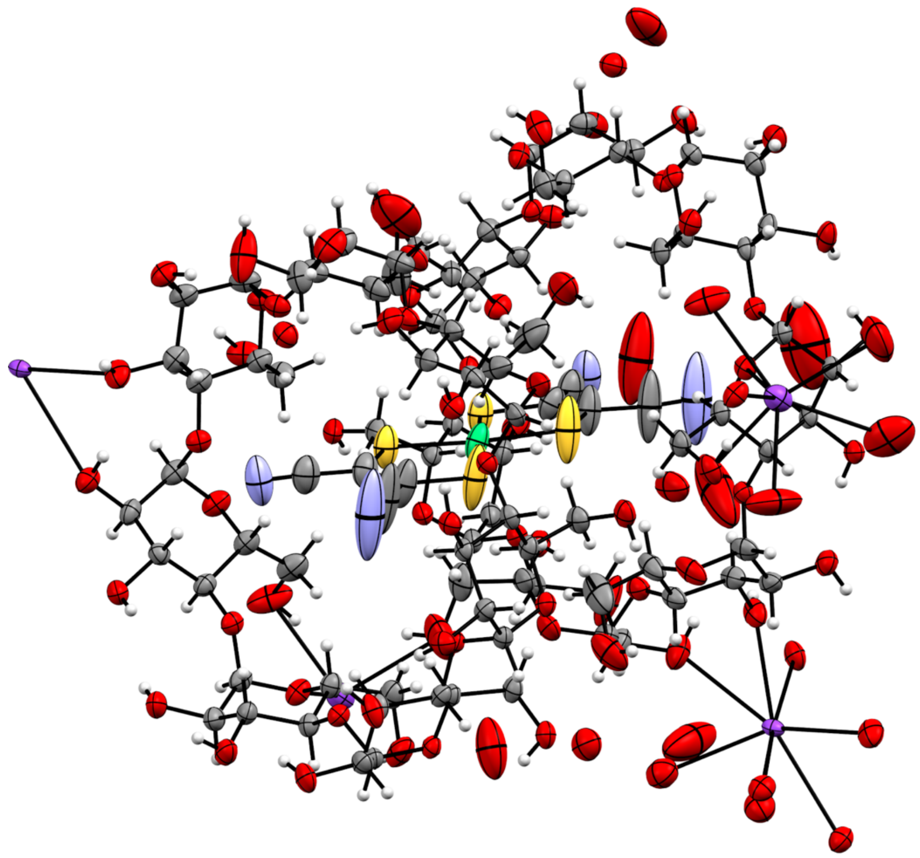

Fig. S1 Crystal structure of the inclusion complex **2**·20H_2O (light green, Ni; yellow, S; gray, C; blue, N; red, O; purple, K; white, H).

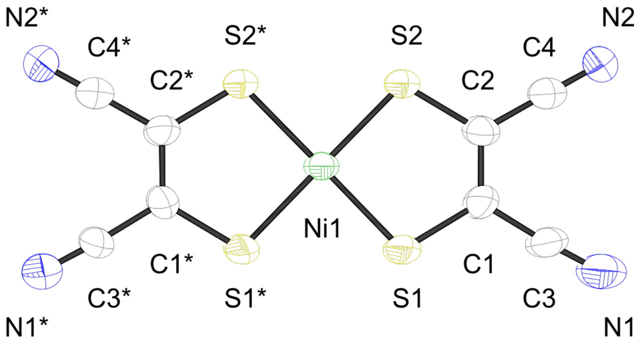

Fig. S2 Numbering scheme for the $[Ni(mnt)_2]^{2-}$.* indicates the equivalent atoms generated by the symmetry operators (−x + 1, y, −z).

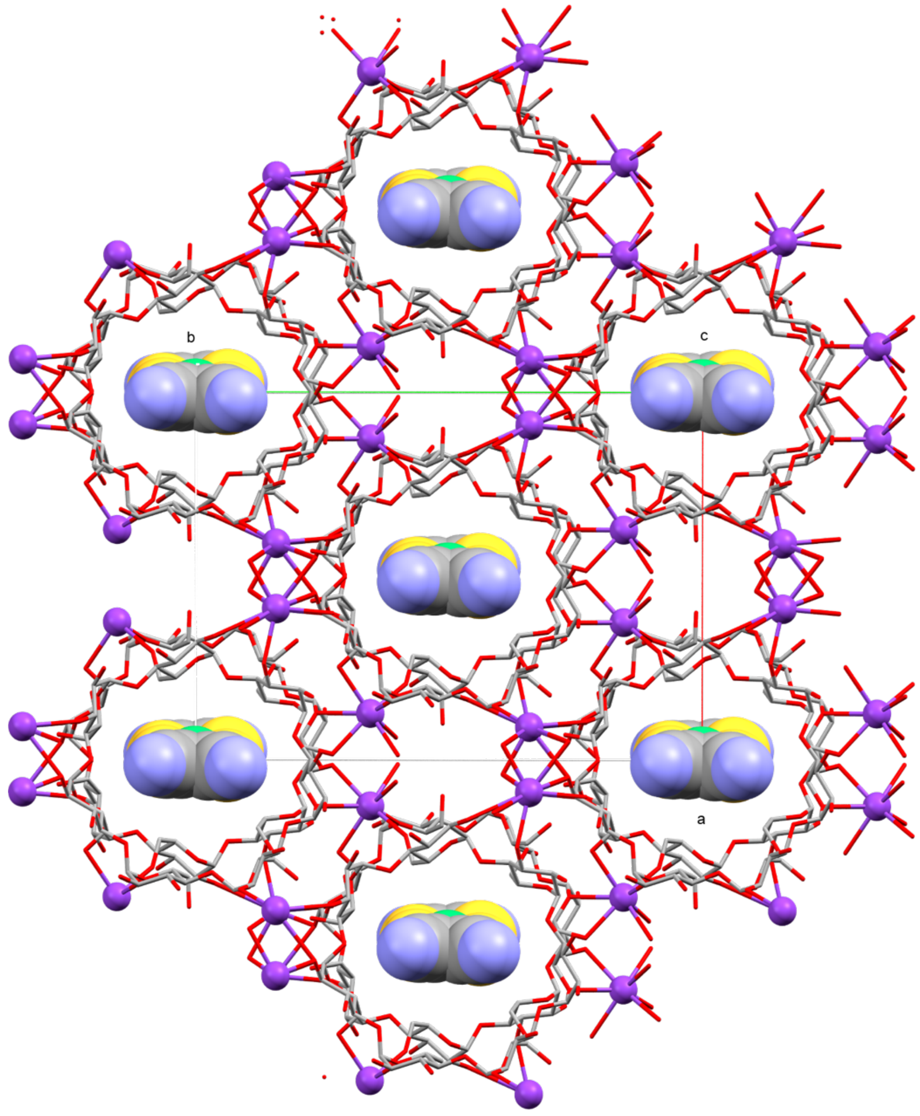

Fig. S3 Molecular packing of the inclusion complex **2**·20H_2O as viewed from the c-axis direction. $[Ni(mnt)_2]^{2-}$, CPK model; β-CD and water, capped sticks model; counter cations, ball and stick model (light green, Ni; yellow, S; gray, C; blue, N; red, O; purple, K; white, H). Water molecules not connected to counter anions and protons are omitted for clarity.

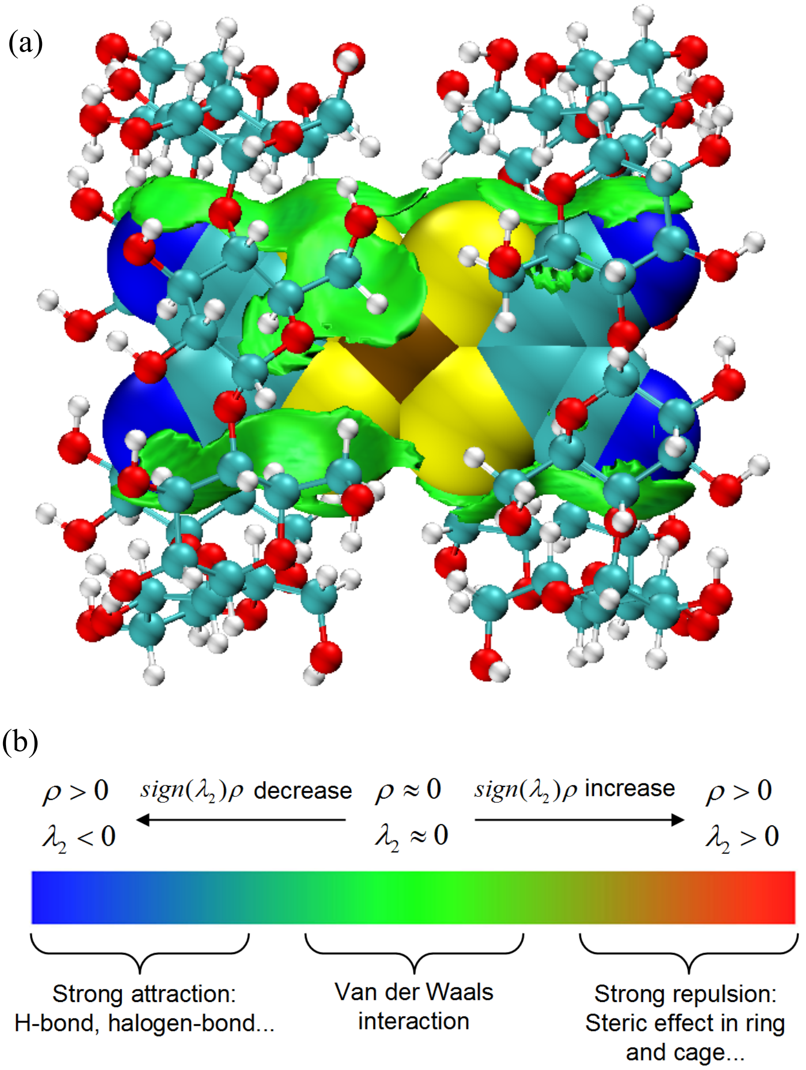

Fig. S4 (a) The isosurface plot of δg_{inter} for the weak interaction between β-CDs and cyano groups of the anion guest complex in the inclusion complex, $-0.5 < sign(\lambda_2)\rho < 0.05$ a.u. The isovalue of δg_{inter} is 0.001. (b) Interpretation of coloring method of mapped function $sign(\lambda_2)\rho$ in IGMH.

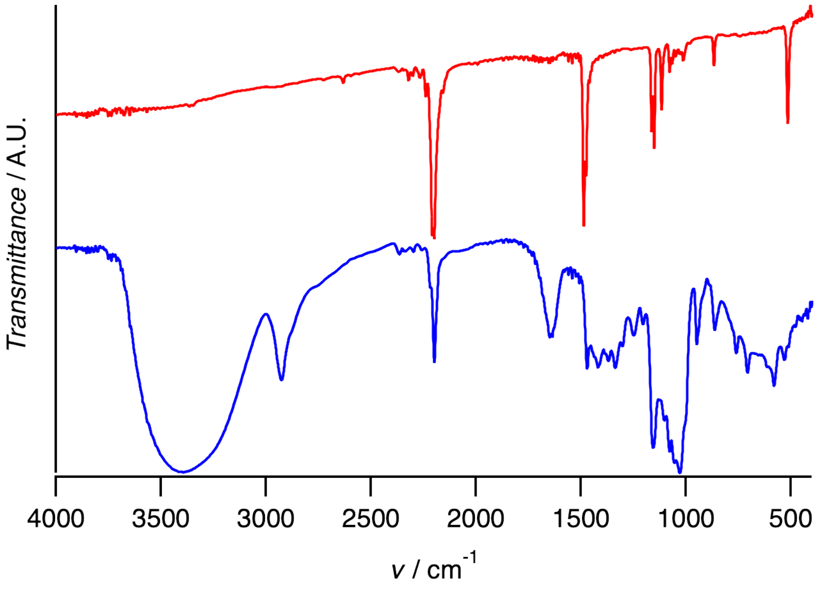

Fig. S5 IR spectra of the free Ni complex **1** (red) and inclusion complex **2** (blue). Selected ν_{max}/cm^{-1} = 2208 (CN): 2198 (CN) for **1** and 2198 (CN) for **2**.

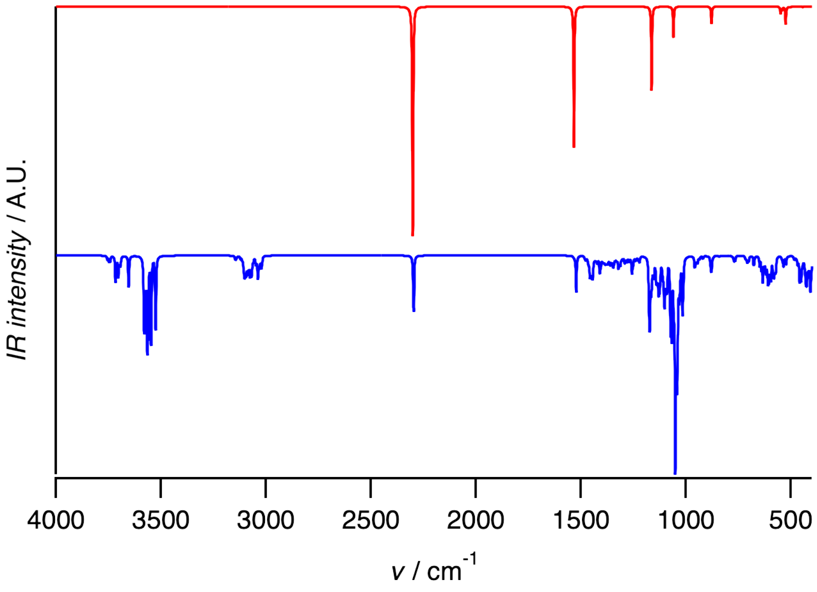

Fig. S6 Simulated IR spectra of the free Ni complex **1** with a square planar geometry (red) and inclusion complex **2** (blue). Selected ν_{max}/cm^{-1} = 2301 (CN) for **1** and 2296 (CN) for **2**.

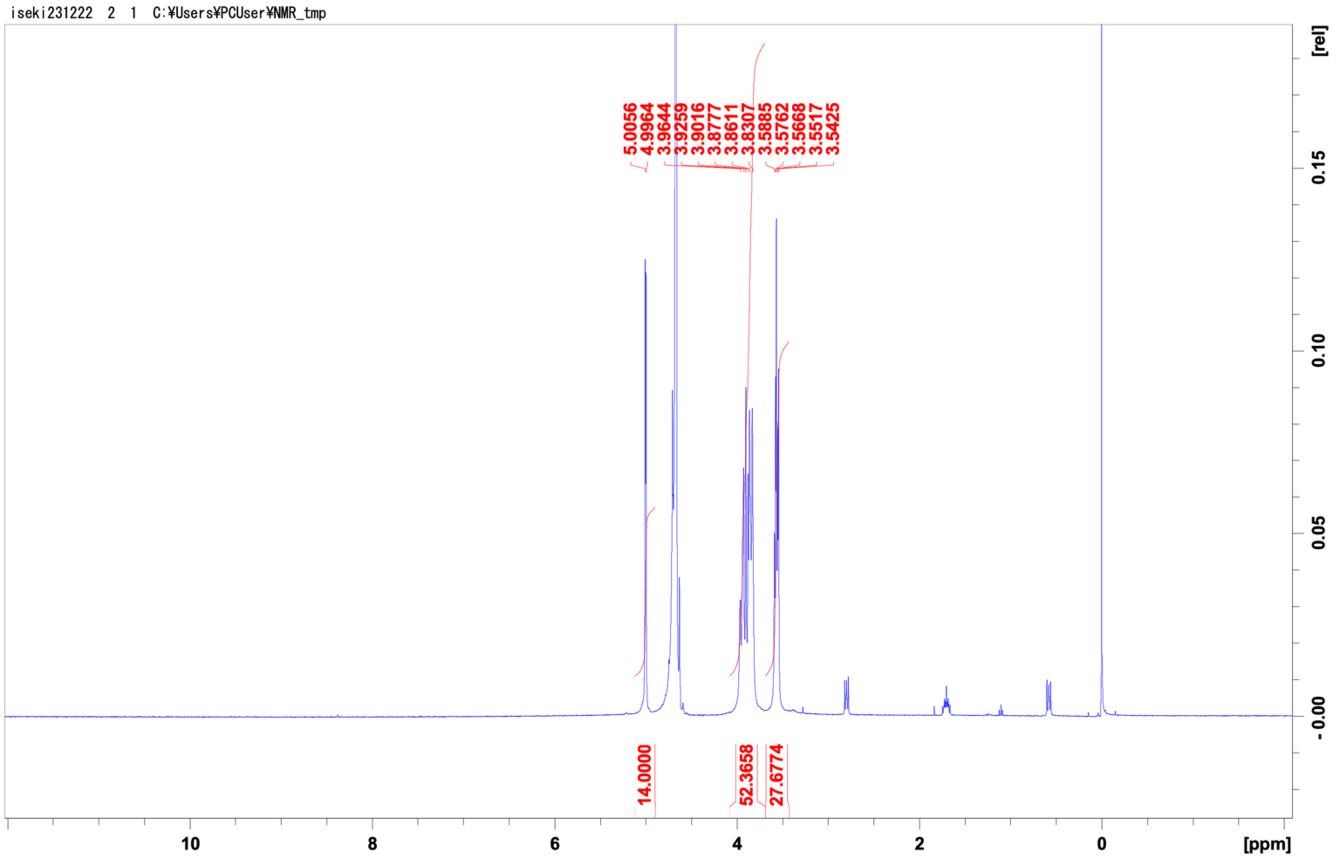

Fig. S7 ^{1}H NMR spectrum of the inclusion complex **2** in D_2O. The concentration was 1×10^{-3} mol dm^{-3}.

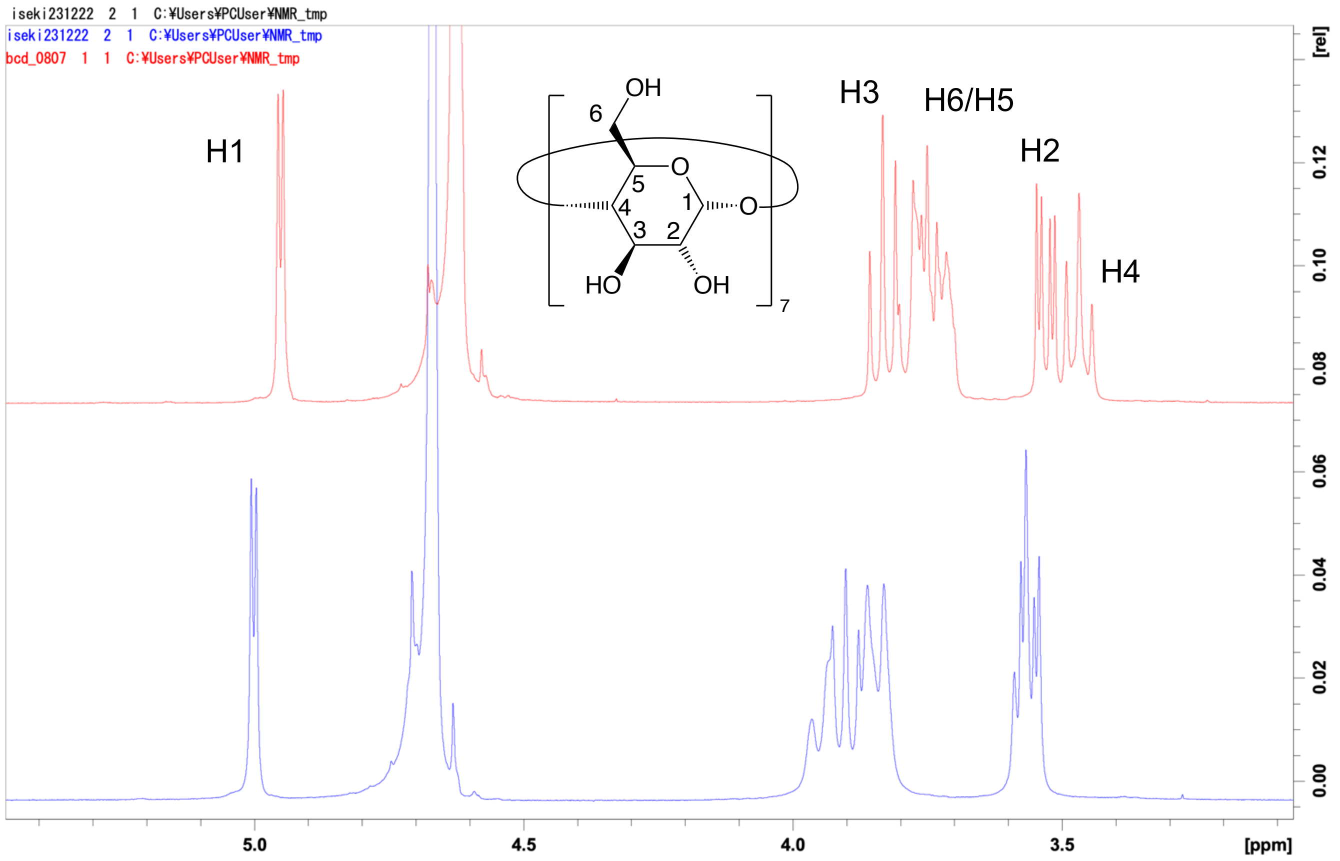

Fig. S8 ^{1}H NMR spectra of β-CD (red) and the inclusion complex **2** (blue) in D_2O.

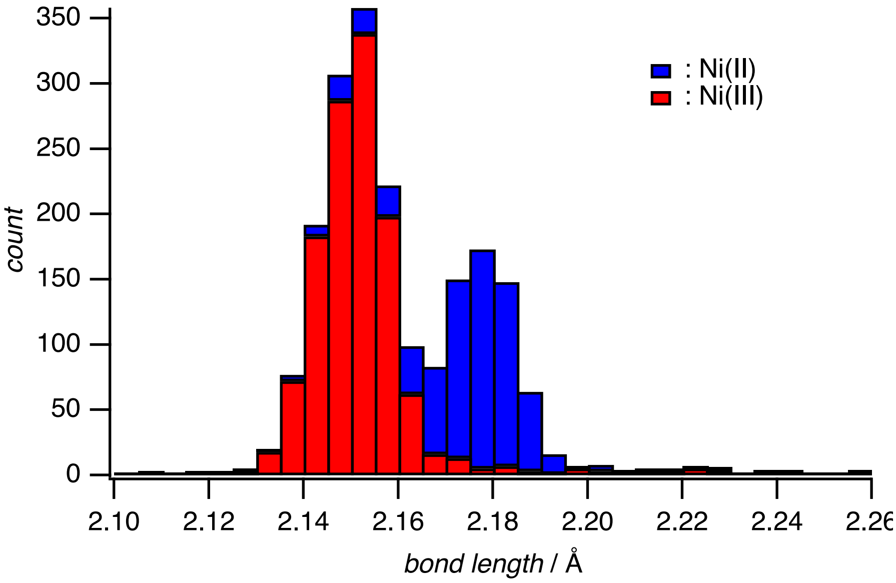

Fig. S9 Histogram of the Ni–S bond distances in $[Ni(mnt)_2]^{n-}$ determined from a search in the CCDC database (updated June 2024)

CDS refcodes of $[Ni^{II}(mnt)_2]^{2-}$ for Fig. S7: AFUXIK, AGUZUX, ARUMAD, ASEFEM, ASOYOW, ASOZAJ, AWOSUB, BAHMEE, BAJFOK, BAJFUQ, BOZXUL, CASNUH, COPFUI, DAFWOX, DAFWUD, DAGTEN, DAKLOQ, DEGLOR, DERXUU, DIHWOI, DIPZIP, DOZSUH, DOZTAO, DUHTEF, EBOZAX, ECPMTN, EGEPEL, ERIQIG, ESEROJ, ETONEH, ETONIL, ETULAH, FOSREM, FUZBUY, GAKGAB, GIWREL, GUPZOG, GUPZOG01, GUPZUM, GUQBAV, GUQBEZ, GUQBID, GUTWOH, GUVCIK, GUVDAD, HAMPUH, HAVREC, HIMWAB, HOMHIC, HOMNOQ, HOMNOQ01, HUKTIQ, HUNQOX, ICUBEO, IDAQOV, IFUMEC, IGOYIM, IPALUG, IWOXIB, JAKNOB, JAKNUH, JUHQIO, KAKLUD, KANYOP, KEHNUJ, KEHNUJ01, KIGXIH, KIKBUB, KOFVIL, KOFVOR, KOFVUX, KOFWAE, KOFWEI, LAHYIC, LAVGEW, LEBPOY, LEHVEB, LEHVEB01, LEHVEB02, LEWFEA, LEWFIE, LIKLOI, MDTNIP, MIQXIU, MNTNBA, MOQCEB, NADVUK, NIQLUV, NOHZUG, OKUNUD05, OPALUN, OXEBAV, PAGMNU, PEHNEW, PIWXUP, PULRIZ, PULROF, PULSAS, QAHVEC, QAQCER, QEBRUM, QEVGAZ, QOQYIF, RAFWIF, REJHIA, RELVEL, RIGSOQ, SEKWEL, SEKWIP, SOZPOO, TEFGAQ, TEFGEU, TESJUY, TICCUD, TIQDAC, TMASNI, TMDMNI, VARQAH, VAVGOP, VICDAM, VIYLAS, VIYLAS01, VUNQOL, VUQXAH, WEBFOA,

WEMNUX, WENFED, WENXUJ, WEYBUY, WIHJON, WINDAZ, WINGIK, WIPDOP, WONHUC, WUXNEJ, WUXNIN, XAVCAY, XAVCEC, XEHKOL, XIBQAB, XIRGUB, XIVXAD, XIVXAD01, XIVXAD02, XIVXAD03, XIVXAD04, XOJWUQ, XULRUS, YATRES, YAZXON, YIZBEP, YOBMAD, YUFCOR, YUFHUC, ZIGJUX, ZIGKAE, ZUZQAM, ZUZQIU

CDS refcodes of $[Ni^{III}(mnt)_2]^-$ for Fig. S7: ACATOO, ACATUU, ACAVAC, AGETIQ, AGUZOR, AGUZOR01, AGUZOR02, AHABAM, AHABAM01, AHABAM02, AJUHES, ASEHIQ, ASEHIQ01, ASEHIQ02, BAHMII, BEVHIU, BOFQUI, BOFWAU, BOFWUO, BOFZEB, BOPFOD, BUBBAD, BUBBEH, BUBBEH01, BUZBEE, BUZROE, BUZRUK, CAJNUW, CAKCEW, DAHLII, DAXRAW, DEBCIW, DEHXEV, DELROC, DIHXAV, DUHTUV, DUKZOZ, DUKZOZ01, DUKZOZ02, EAMNTN, EAMNTN01, ECUYAD, EDARAD, EDAREH, ETONEH, FENCOR, FETBOW, FITYEN, FOQXOZ, FOQXUF, FOQYAM, FOQYEQ, FORBUK, FOTTIR, FUDXEH, FUDXEH01, FUDXEH02, FUDXEH03, FUDXEH04, GALRER, GELSUL, GERLEU, GIRHOG, GOTGOL, GUBJUJ, GUBKAQ, GUVCOQ, GUVCUW, GUVDEH, HAVRIG, HAXPEC, HETHEU, HOMHEY, HOQDIB, HOQDOH, HOZVEZ, HUFKEY, HUKTOW, HUMXOD, IDAZUK, IDAZUK01, IDAZUK02, IGOJAP, IGOJAP02, IGOYIM01, IHAQEN, IHAQEN01, INAYEB, JARMAR, JUQDUU, KACNEI, KAYKAW, KITJII, KITJOO, KITJUU, KITKAB, KITKEF, KITKIJ, KUFCAQ, KUFCAQ01, KUFCAQ02, KUFCEU, KUFCEU01, LAMXEF, LEHCUX, LEHDAE, LEHTAU, LEXJEF, MEMVOQ, MEMVUW, MEMWAD, MIJHUJ, MUTZOR, NADVUK, NASQOO, OCIGAK, ODIKIW, OGEHOX, OGEZIL, OGEZOR, OGINOJ, OKOVEQ, OKOVIU, OKUNUD, OKUNUD01, OKUNUD02, PAKDAI, PAXJII, PECFAF, PIMQOR, PIMROS, PMPESN, POQVIB, PORYOM, PORYUS, POTTOH, POYSIG, POYSOM, QADTEY, QADTEY01, QEQQIN, QEQQOT, QUKSAS01, QUKSAS02, QUKSAS05, QUKSAS06, QUKSAS07, QUPNIY, RADXAW, RAMTAB, RAMTEF, RAWWOB, RECDEJ, RECDEJ01, RECDEJ02, RECDEJ03, REHMIB, REJHIA, RIGMOL, RIGMOL01, RIGMOL02, RIGMOL03, RIGMOL04, RIGMOL05, RIGMOL06, RIGMOL07, RIGMOL08, RIGMOL09, RIGMOL10, SELPAB, SELPEF, SIXJAL, SIXJEP, SIZNUL, SIZNUL01,

SIZNUL02, SIZNUL03, SIZNUL04, SUZTUC, TAFQEY, TECRAW, TROPNJ, UCUHEG, UPIZID, VATTIU, VAXWUO, VEKCOE, VEKCUK, VEWBEF, VEZBOS, VEZCAF, VEZCAF01, VEZCAF02, VEZCEJ, VIDHIA, VIDHIA02, VOSTOO, VOTVIM, WACNEW, WAMHIC01, WAMHIC02, WOPFEO, WOPMUL, WOPNEW, WOPNIA, WOPNOG, WOPRID, WOPROJ, WOQPEZ, XEGGOG, XEGGUM, XEHQUX, XEHQUX01, XEHQUX02, XEHQUX03, XEHQUX04, XEHQUX05, XEHQUX06, XEHQUX07, XEHQUX08, XEHQUX09, XEHQUX10, XIDMAA, XIDMAA01, XIJYEU01, XIJYEU02, XUCVOH, XUCVUN, XULSAZ, XULZIO, XULZOU, XUNDOZ, XUNDOZ01, YAFCAL, YAVXAV, YAVXAV01, YAVXAV02, YAVXAV03, YIZBIT, YOLJEQ, YOLJIU, YOLJOA, ZETYEF, ZICHIE, ZICHOK, ZIRSEA, ZIRSIE

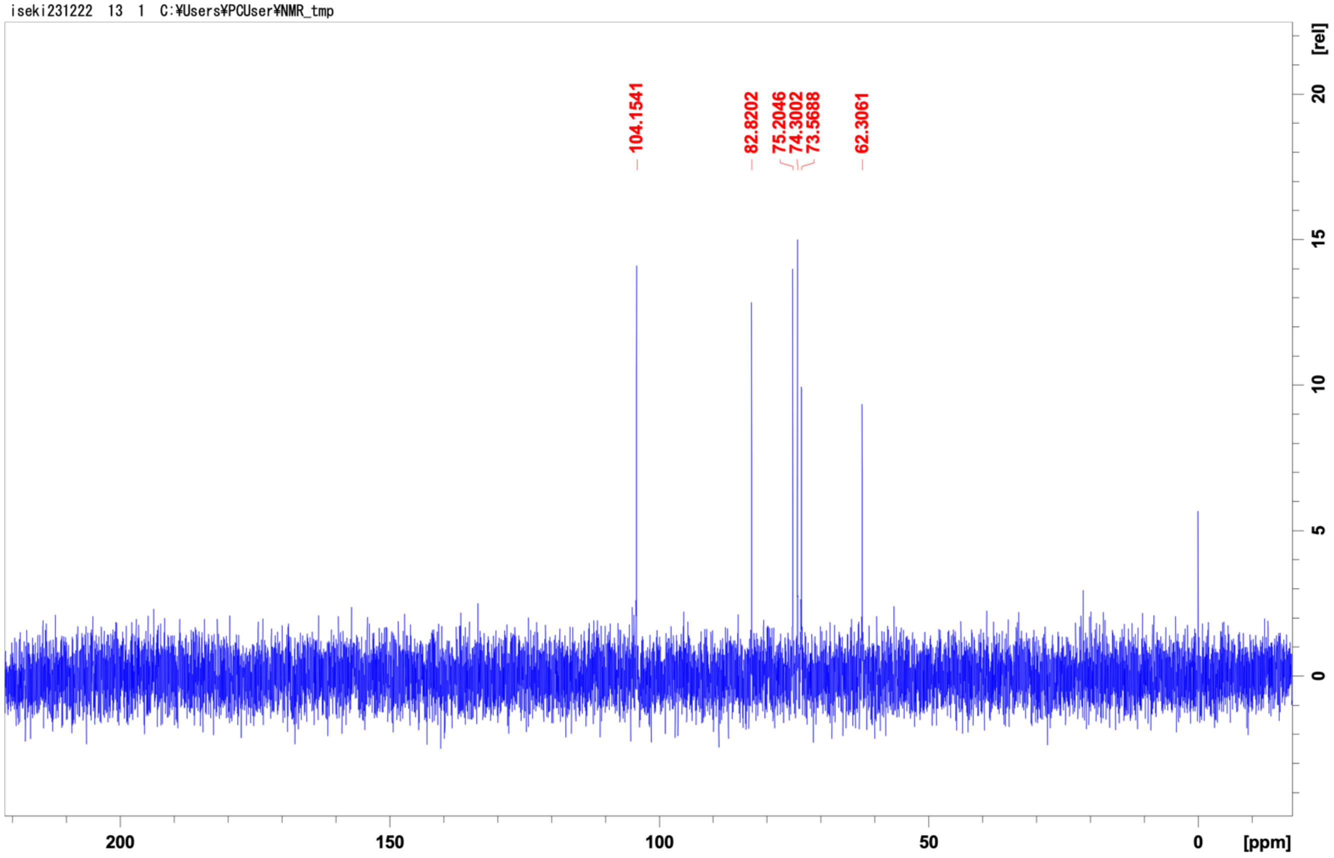

Fig. S10 ^{13}C NMR spectrum of the inclusion complex **2** in D_2O. The concentration was 1×10^{-3} mol dm^{-3}.

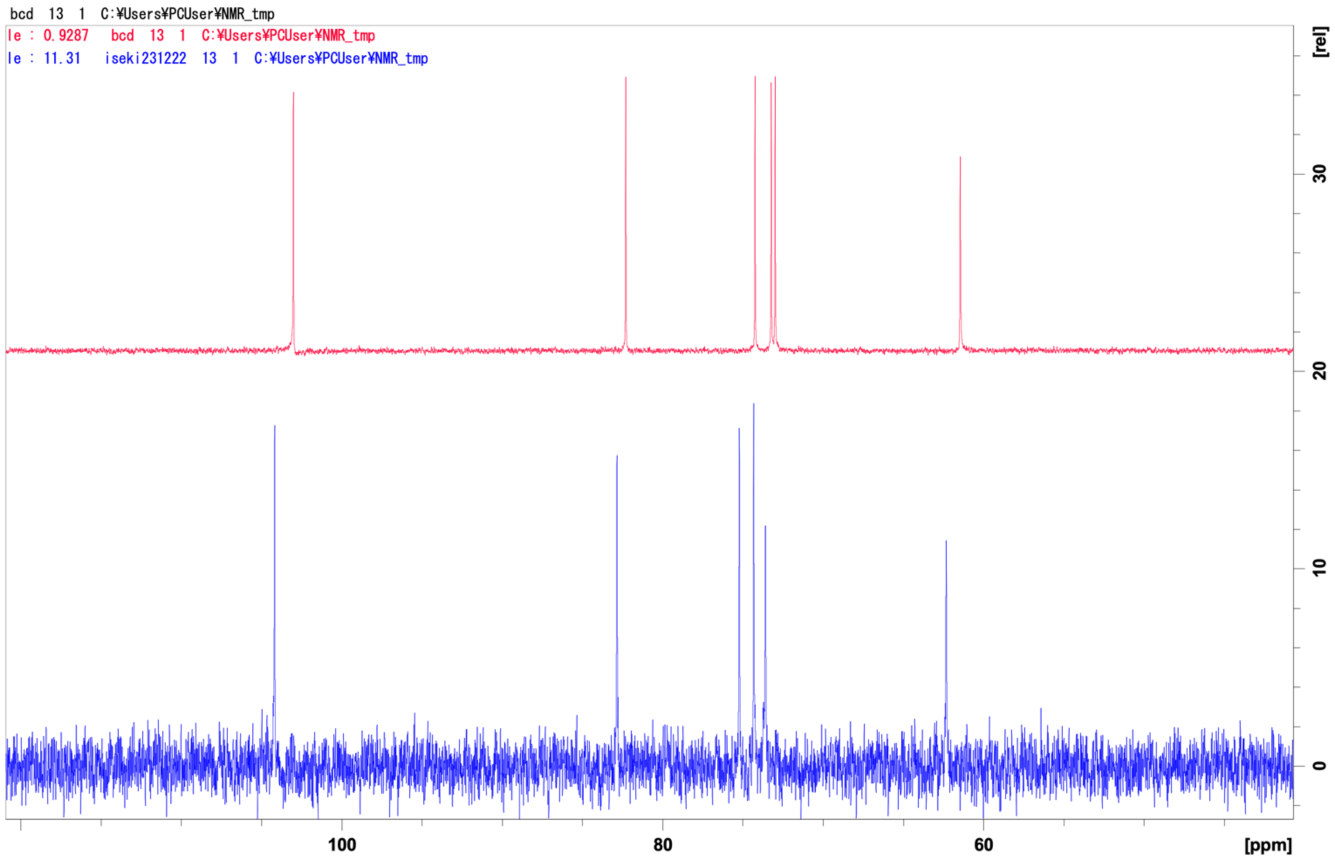

Fig. S11 ^{13}C NMR spectra of β-CD (red) and the inclusion complex **2** (blue) in D_2O.

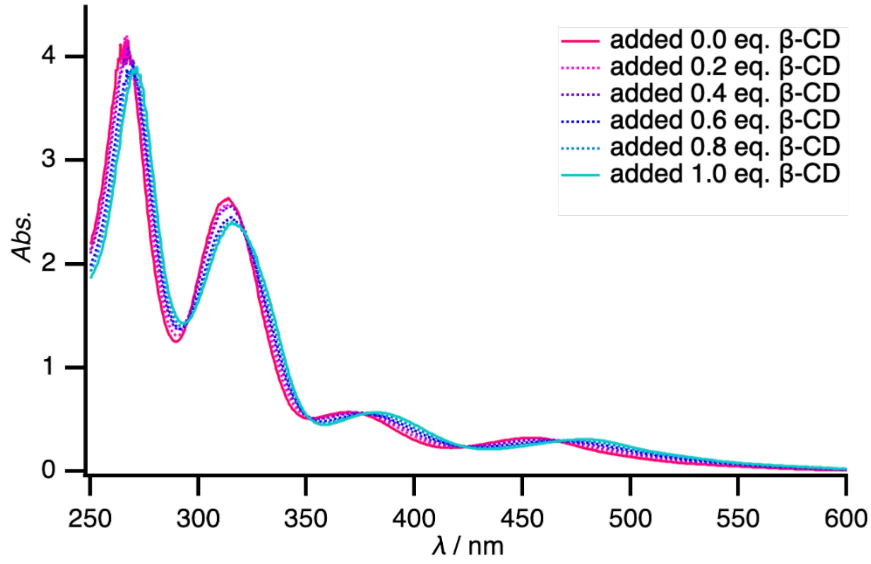

Fig. S12 UV–Vis spectrum of the free Ni complex **1** with β-CD (0.0–1.0 eq.). The conditions were the same as those depicted in Fig. 5.

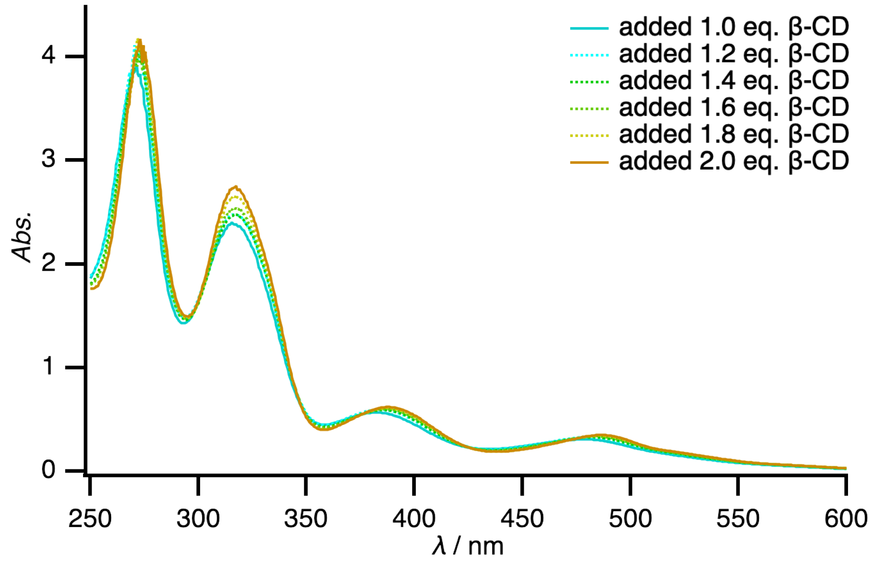

Fig. S13 UV–Vis spectrum of the free Ni complex **1** with β-CD (1.0–2.0 eq.). The conditions were the same as those depicted in Fig. 5.

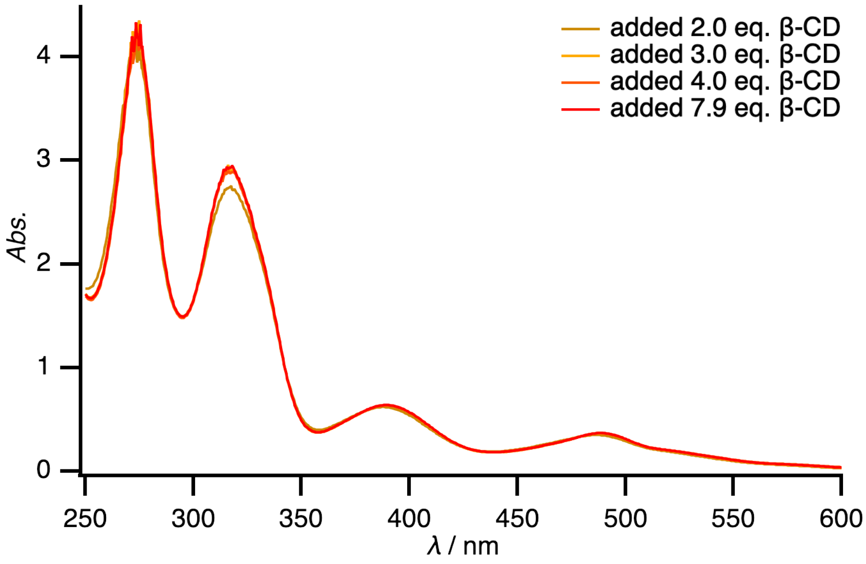

Fig. S14 UV–Vis spectrum of the free Ni complex **1** with β-CD (2.0–7.9 eq.). The conditions were the same as those depicted in Fig. 5.

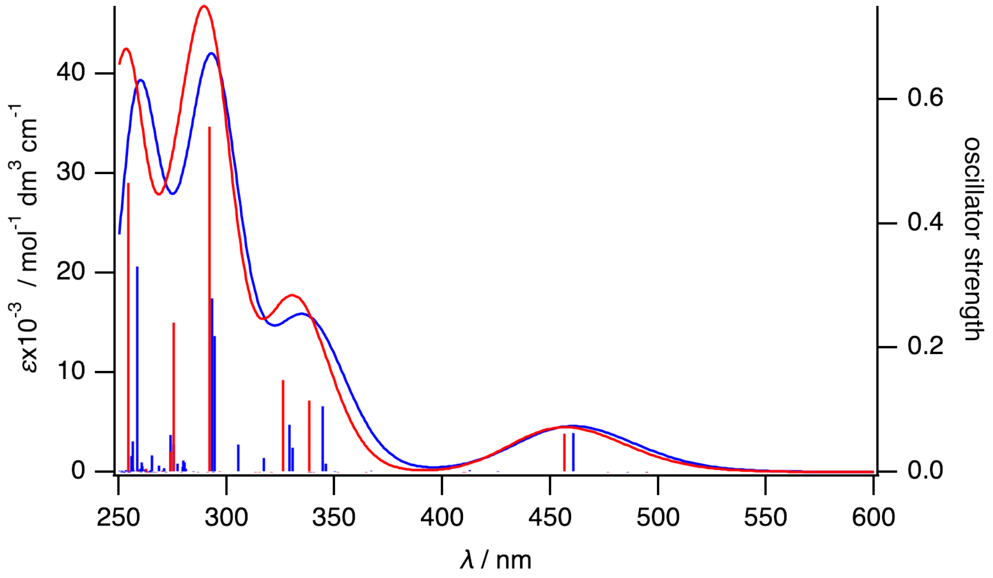

Fig. S15 Simulated UV–Vis spectra of the free Ni complex **1** with a square-planar geometry (red) and inclusion complex **2** (blue). The solid line indicates the simulated spectra (left scale), and the vertical lines indicate the oscillator strength (right scale). The solvent effect of water was applied.

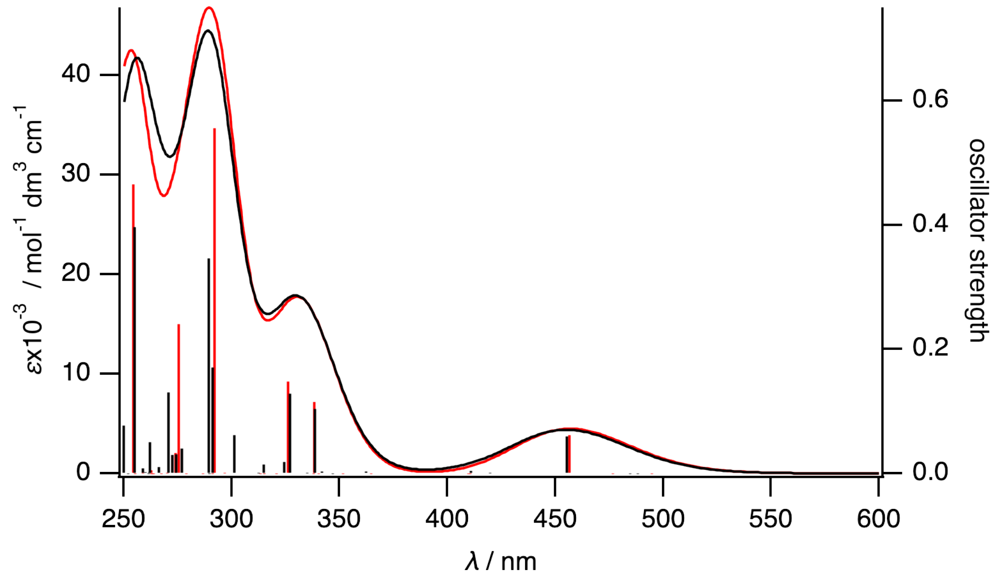

Fig. S16 Simulated UV–Vis spectra of the free Ni complex **1** with a square-planar geometry (red) and with a slightly distorted square-planar geometry (black). The solid line indicates the simulated spectra (left scale), and the vertical lines indicate the oscillator strength (right scale). The solvent effect of water was applied.

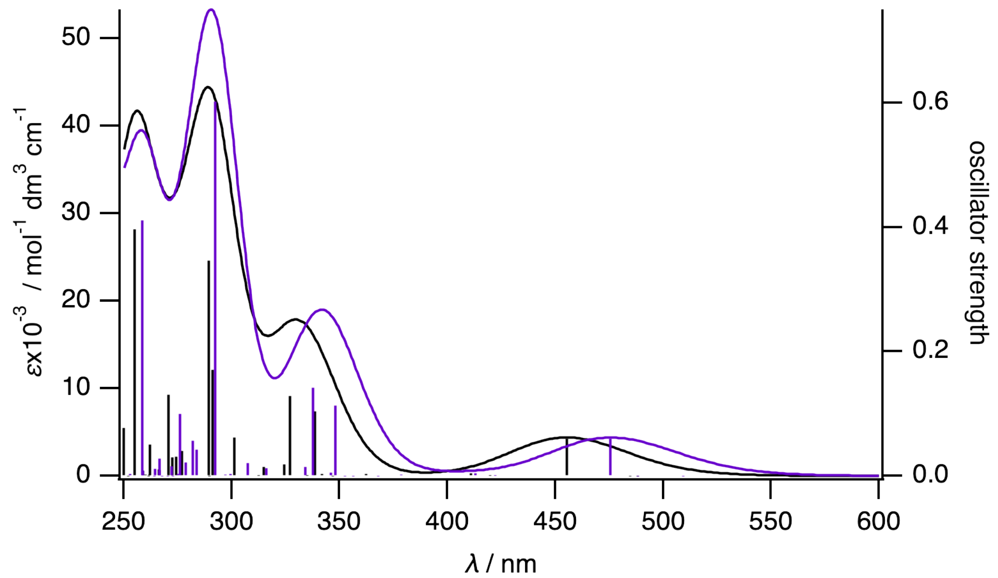

Fig. S17 Simulated UV–Vis spectra of the free Ni complex **1** with a slightly distorted square-planar geometry in water (black) and in heptane (purple). The solid line indicates the simulated spectra (left scale), and the vertical lines indicate the oscillator strength (right scale).

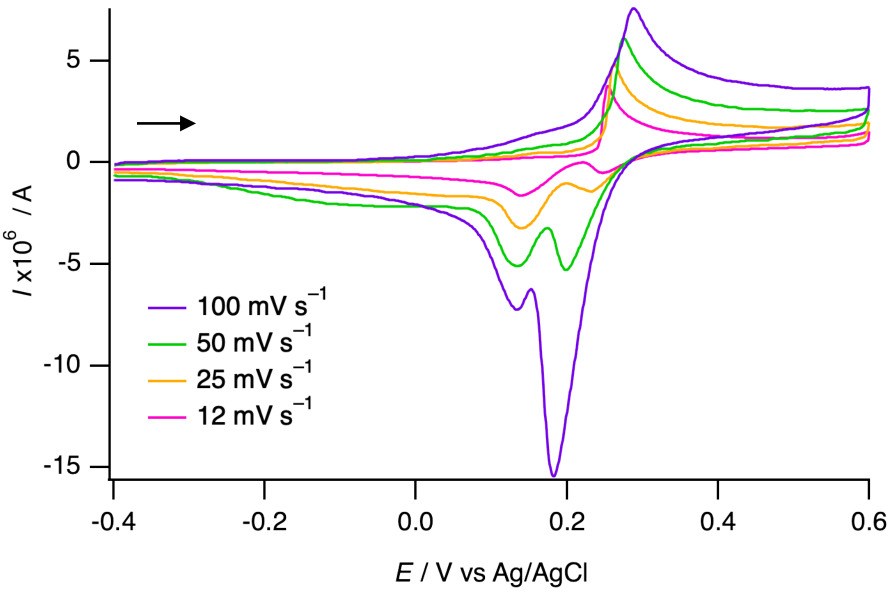

Fig. S18 Cyclic voltammograms of the free Ni complex **1** at different scan rates. The conditions were the same as those depicted in Fig. 6, except for the scan rate.

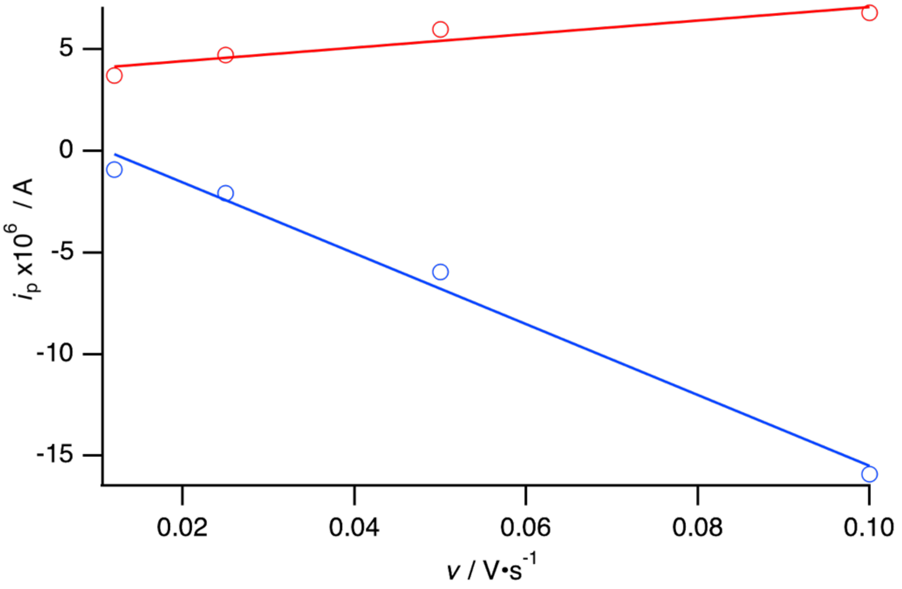

Fig. S19 Plot of peak current versus scan rate for the free Ni complex **1.** The red open circles indicate the anodic peak current i_{pa}. The blue open circles indicate the cathodic peak current i_{pc1}. The solid line shows the linear curve fitted to the peak currents.

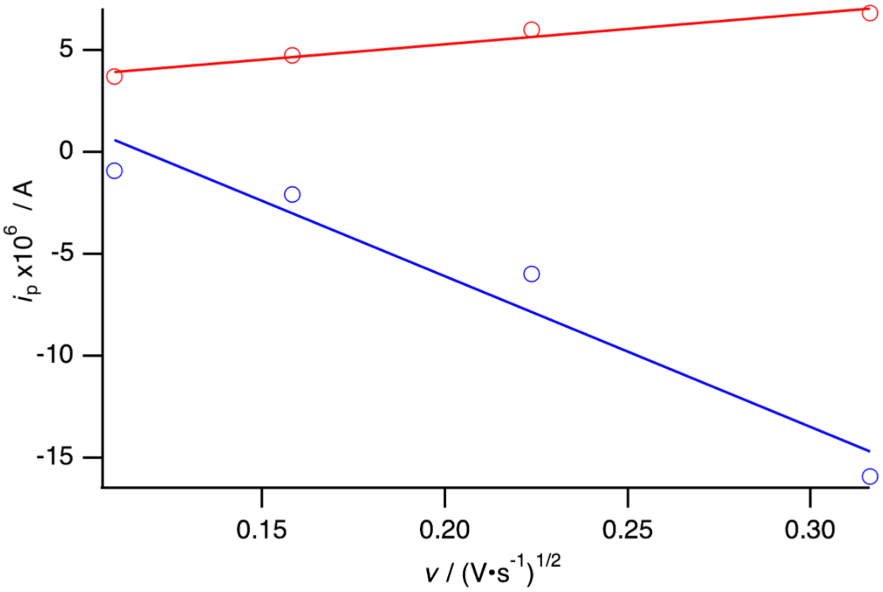

Fig. S20 Plot of peak current versus the square root of the scan rate for the free Ni complex **1.** The red open circles represent the anodic peak current i_{pa}. The blue open circles indicate the cathodic peak current i_{pc1}. The solid line indicates the linear fit to the peak currents.

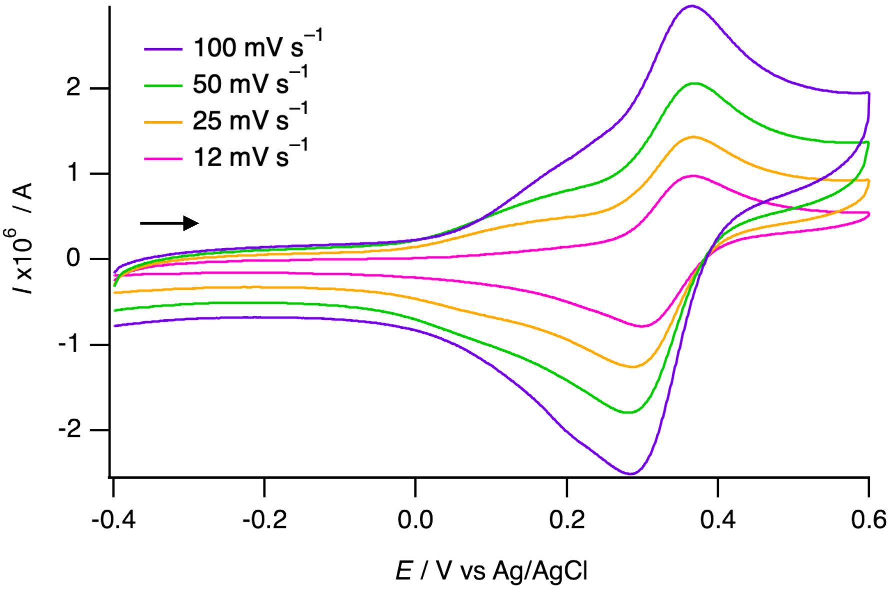

Fig. S21 Cyclic voltammograms of the inclusion complex **2** at various scan rates. The conditions were the same as those depicted in Fig. 6, except for the scan rate.

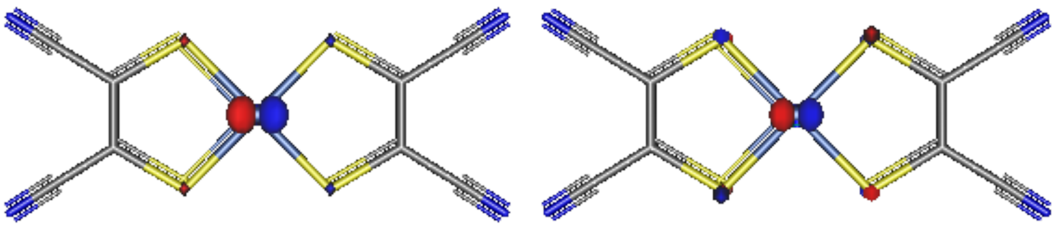

Fig. S22 Calculated molecular orbital. The SOMO(β) in the one-electron-oxidized state of $[Ni(mnt)_2]^{2-}$ is shown on the left. The HOMO in the isolated state of $[Ni(mnt)_2]^{2-}$ is shown on the right.

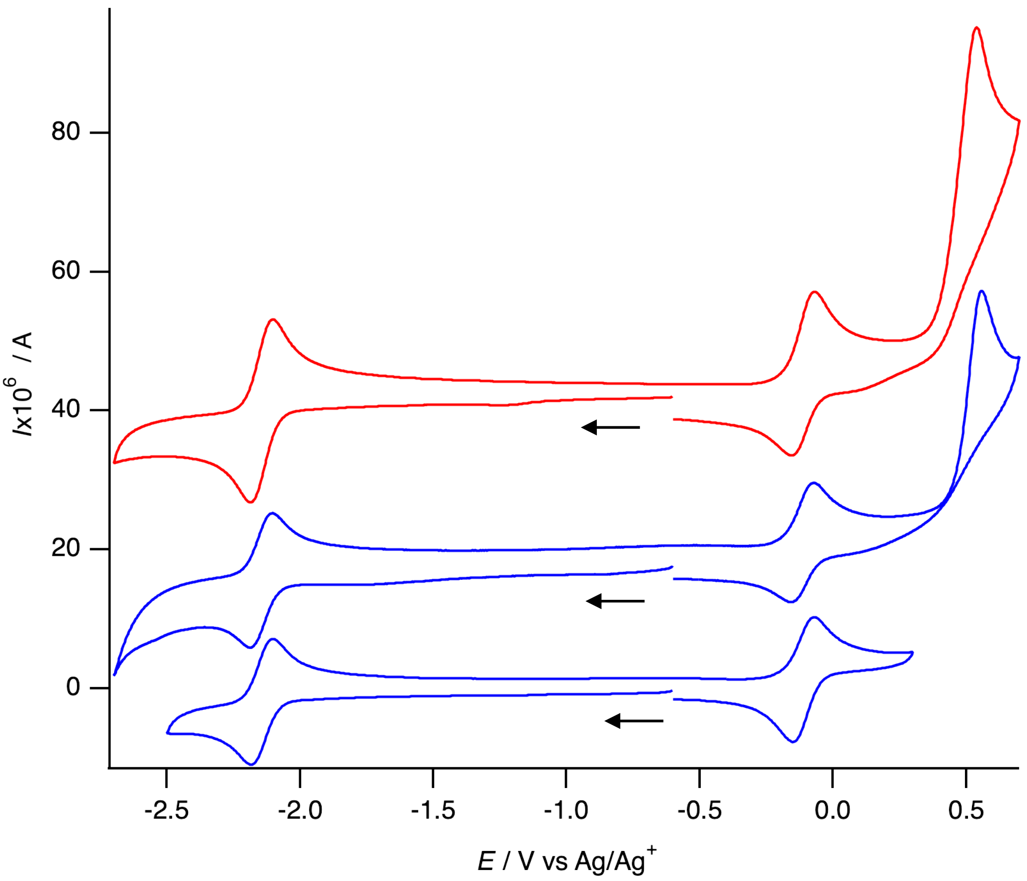

Fig. S23 Cyclic voltammograms of the free Ni complex **1** (red) and inclusion complex **2** (blue) in 0.1 mol dm^{-3} tetrabutylammonium hexafluorophosphate/N,N-dimethylformamide. [complex] = 1×10^{-3} mol dm^{-3}.

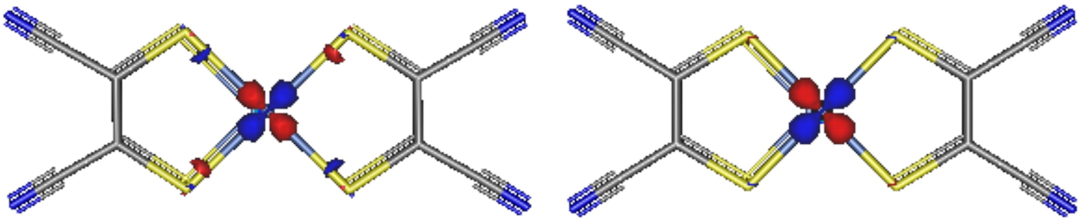

Fig. S24 Calculated molecular orbital. SOMO(α) in the one-electron-reduced state of $[Ni(mnt)_2]^{2-}$ is shown on the left. The LUMO at the isolated state of $[Ni(mnt)_2]^{2-}$ is shown on the right.

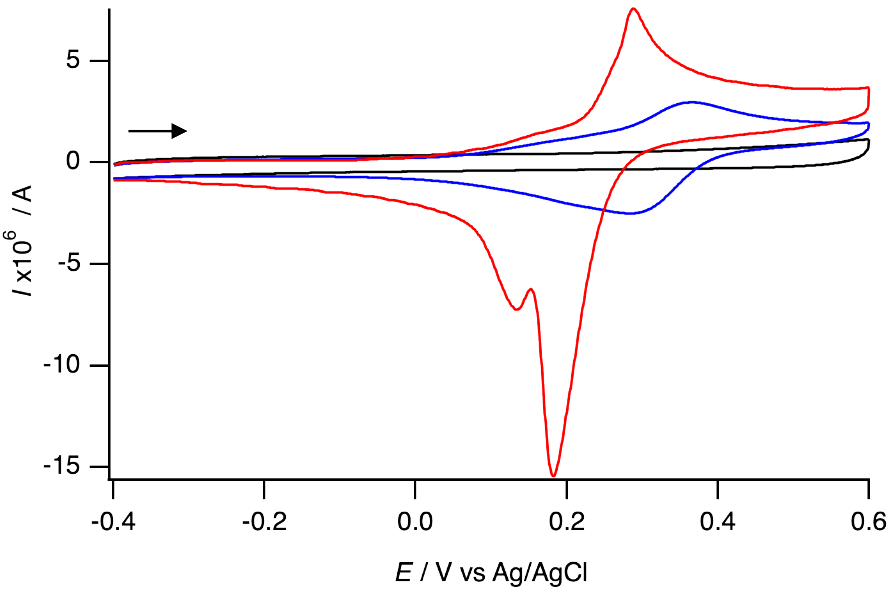

Fig. S25 Cyclic voltammograms of the free Ni complex **1** (red), inclusion complex **2** (blue), and baseline (black). The conditions were the same as those depicted in Fig. 6.

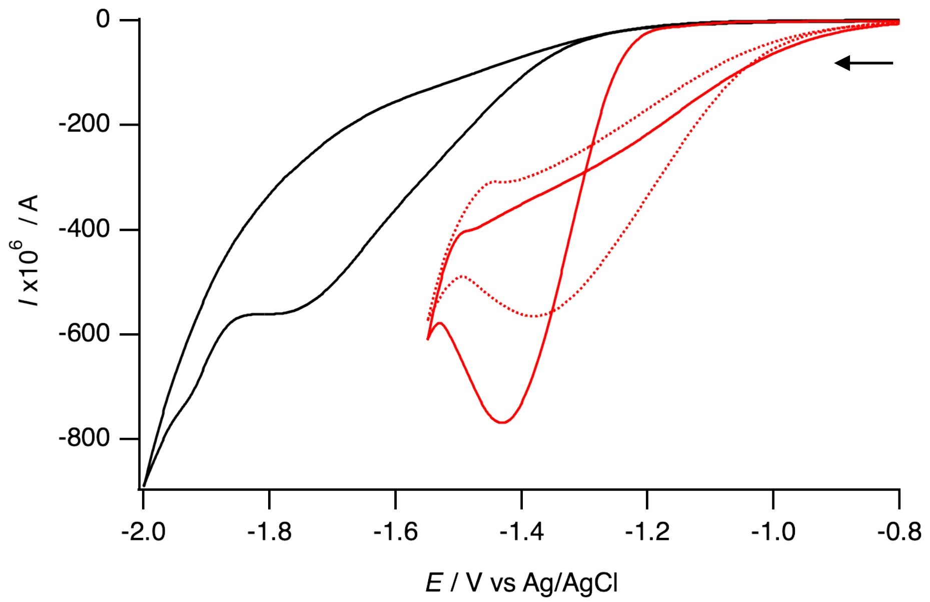

Fig. S26 Cyclic voltammograms of the free Ni complex **1** (red solid line), “rinse test” (red dotted line), and baseline (black). The conditions were the same as those depicted in Fig. 7, except for scan range.

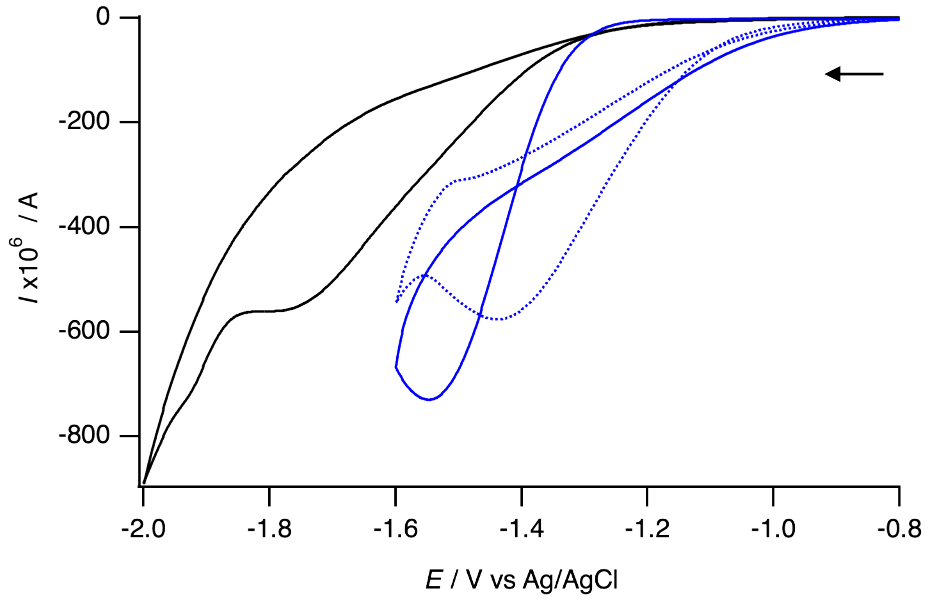

Fig. S27 Cyclic voltammograms of the inclusion complex **2** (blue solid line), “rinse test” (blue dotted line), and baseline (black). The conditions were the same as those depicted in Fig. 7, except for scan range.

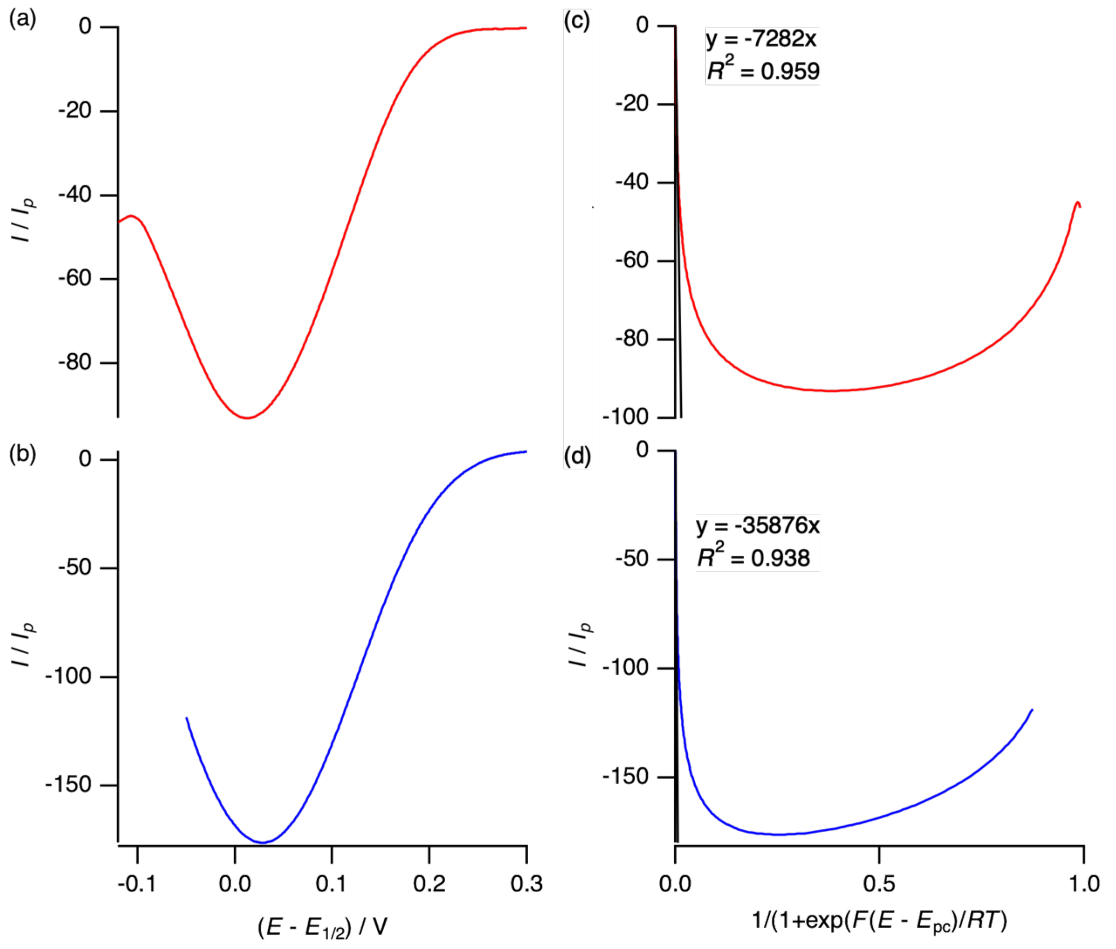

Fig. S28 Left: Baseline-corrected catalytic behavior of the (a) free Ni complex **1** and (b) inclusion complex **2**. The E_{pc} of the catalytic reaction and i_{pa} of the Ni(II)/Ni(III) redox couple were used instead of $E_{1/2}$ and I_p, respectively. The conditions were the same as those depicted in Fig. 7.

Right: Foot-of-the-wave analysis of the (c) free Ni complex **1** and (d) inclusion complex **2**. The black line represents the fitting of the electrocatalytic responses that deviate from the ideal behavior.

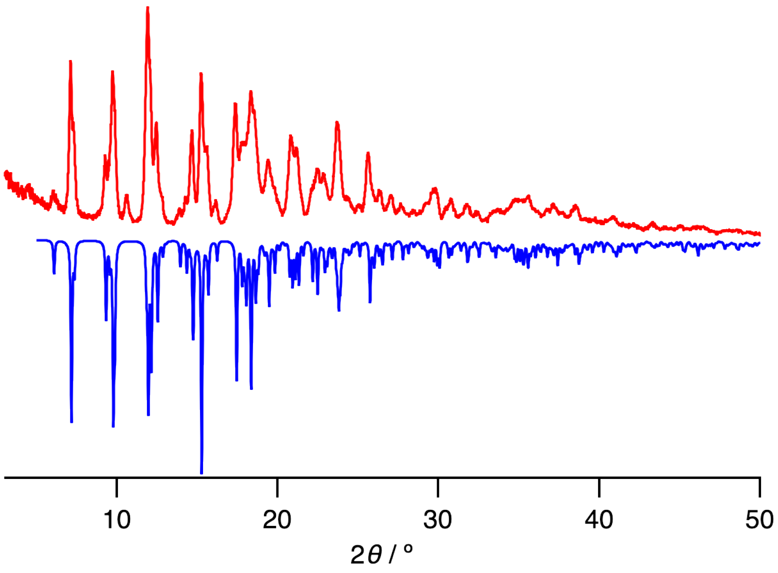

Fig. S29 PXRD patterns of the inclusion complex **2**. The red pattern corresponds to the bulk sample after exposure to ambient atmosphere overnight, while the blue pattern is simulated.

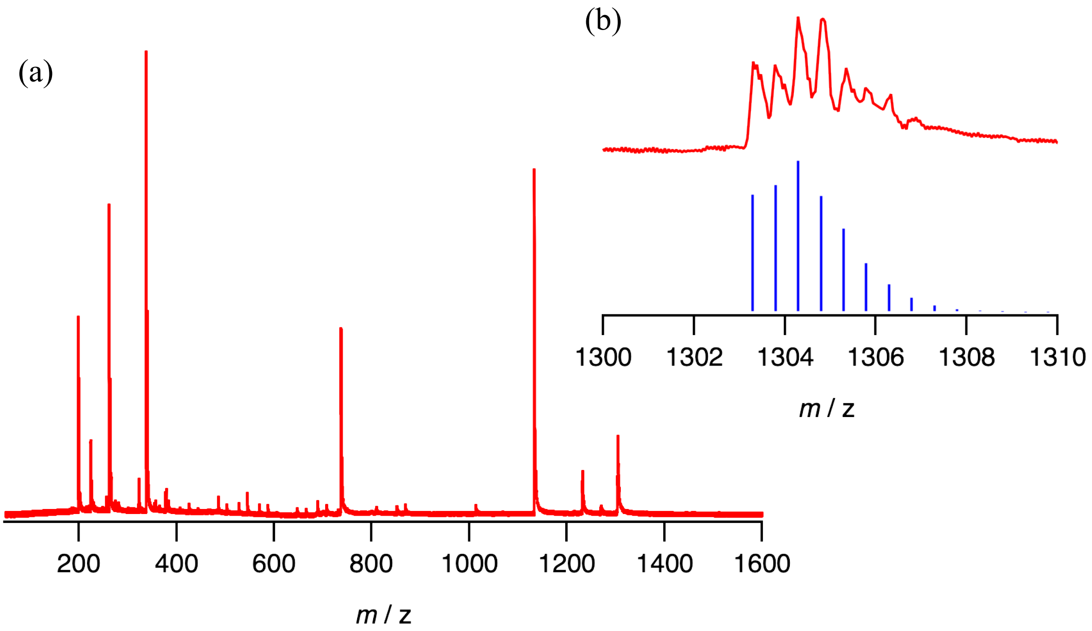

Fig. S30 (a) ESI-mass spectrum of the inclusion complex **1** in the anion mode. (b) A magnified view around *m*/z = 1305. The red pattern was observed and the blue pattern was simulated for $\{[Ni(mnt)_2]@(\beta\text{-}CD)_2\}^{2-}$.

• Parameters of the curve-fitting simulation performed for the association constants (inset of Figure 5)

Program: SPANA for spectral data analysis (Version 5.5.79)
Least Square optimization at 333, 360, 398, 447 and 490 nm
Model: Sequential 1:2 Complex (AB2) Formation Model
[A]<constant> titrated with [B]
[A] = 0.000998475

Optimized Parameters:
Iteration: 9 , R: .9992899 , AIC: −400.1249
K_1 = 11156.25 (sd: 7118.613)
K_2 = 17702.64 (sd: 4653.551)
$\Delta\varepsilon_{11}$ = 606.2058 (sd: 87.82194)
$\Delta\varepsilon_{21}$ = 744.0835 (sd: 4.385701)
$\Delta\varepsilon_{12}$ = −107.5137 (sd: 15.33935)
$\Delta\varepsilon_{22}$ = −161.4261 (sd: 3.051344)
$\Delta\varepsilon_{13}$ = 216.1581 (sd: 32.07026)
$\Delta\varepsilon_{23}$ = 269.3259 (sd: 3.189408)
$\Delta\varepsilon_{14}$ = −123.8954 (sd: 23.55431)
$\Delta\varepsilon_{24}$ = −118.975 (sd: 3.021309)
$\Delta\varepsilon_{15}$ = 163.4167 (sd: 26.00643)
$\Delta\varepsilon_{25}$ = 194.1131 (sd: 3.086439)
Residual Square Sum : 1.466321E-03

Table S1 Reversibility of Ni(II)/Ni(III) redox couple of the free Ni complex **1**

scan rate/mV s^{-1}	E_{pa}/V*	i_{pa}/A	E_{pc1}/V*	i_{pc1}/A
100	0.29	6.82×10^{-6}	0.18	1.59×10^{-5}
50	0.28	6.00×10^{-6}	0.20	5.96×10^{-6}
25	0.26	4.73×10^{-6}	0.23	2.08×10^{-6}
12	0.25	3.71×10^{-6}	0.25	9.24×10^{-7}

* Potentials are reported with respect to Ag/AgCl.

Table S2 Reversibility of Ni(II)/Ni(III) redox couple of inclusion complex **2**

scan rate/mV s^{-1}	E_{pa}/V*	i_{pa}/A	E_{pc}/V*	i_{pc}/A
100	0.37	2.59×10^{-6}	0.29	2.62×10^{-6}
50	0.37	1.63×10^{-6}	0.29	1.90×10^{-6}
25	0.37	1.09×10^{-6}	0.29	1.38×10^{-6}
12	0.37	8.49×10^{-7}	0.30	9.18×10^{-7}

* Potentials are reported with respect to Ag/AgCl.

Table S3 Electrochemical behavior of the free Ni complex **1** in *N,N*-dimethylformamide

E_{pa}/V*	i_{pa}/A	E_{pc}/V*	i_{pc}/A	$E_{1/2}$/V*
−2.10	1.27×10^{-5}	−2.18	1.27×10^{-5}	−2.14
−0.07	1.27×10^{-5}	−0.15	7.96×10^{-6}	−0.11
0.54	4.45×10^{-5}			

* Potentials are reported with respect to Ag/Ag^{+} in this study.

Table S4 Catalytic data

	i_{pc}/A	E_{pc}/V*
Free Ni complex **1**	7.61×10^{-4}	−1.43
Rinse test of the free Ni complex **1**	4.56×10^{-4}	−1.37
Inclusion complex **2**	7.26×10^{-4}	−1.55
Rinse test of the inclusion complex **2**	5.08×10^{-4}	−1.43

* Potentials are reported with respect to Ag/AgCl.

i_c (corrected catalytic peak current of complex)

= i_{pc} (catalytic peak current of complex) − i (current of baseline at E_{pc})

i_c (corrected catalytic peak current of rinse test)

= i_{pc} (catalytic peak current of rinse test) − i (current of baseline at E_{pc})

i_{cat} (net catalytic peak current)

= i_c (corrected catalytic peak current of complex)

− i_c (corrected catalytic peak current of rinse test)

i, i_c, i_{pc}, and E_{pc} are the current, cathodic current, cathodic peak current, and cathodic peak potential, respectively.

The baseline currents were 2.99×10^{-5} A at −1.37 V vs. Ag/AgCl, 8.33×10^{-5} A at −1.43 V, and 1.98×10^{-4} A at −1.55 V.

• Free Ni complex **1**

i_c (corrected catalytic peak current of complex) = $7.61 \times 10^{-4} - 8.33 \times 10^{-5} = 6.78 \times 10^{-4}$ A

i_c (corrected catalytic peak current of rinse test) = $4.56 \times 10^{-4} - 2.99 \times 10^{-5} = 4.26 \times 10^{-4}$ A

i_{cat} (net catalytic peak current) = $6.78 \times 10^{-4} - 4.26 \times 10^{-4} = 2.52 \times 10^{-4}$ A

$i_{cat}/i_p = 2.52 \times 10^{-4} / 6.82 \times 10^{-6} = 37.0$

• Inclusion complex **2**

i_c (corrected catalytic peak current of complex) = $7.26 \times 10^{-4} - 1.98 \times 10^{-4} = 5.28 \times 10^{-4}$ A

i_c (corrected catalytic peak current of rinse test) = $5.08 \times 10^{-4} - 8.33 \times 10^{-5} = 4.25 \times 10^{-4}$ A

i_{cat} (net catalytic peak current) = $5.28 \times 10^{-4} - 4.25 \times 10^{-4} = 1.03 \times 10^{-4}$ A

$i_{cat}/i_p = 1.03 \times 10^{-4} / 2.59 \times 10^{-6} = 40.0$

• Inclusion complex **2** vs. free Ni complex **1**

40.0/37.0 = 1.08

Table S5 Electrochemical behavior of inclusion complex **2** in *N,N*-dimethylformamide

E_{pa}/V*	i_{pa}/A	E_{pc}/V*	i_{pc}/A	$E_{1/2}$/V*
−2.10	8.06×10^{-6}	−2.19	9.11×10^{-6}	−2.15
−0.07	8.93×10^{-6}	−0.15	5.54×10^{-6}	−0.11
0.56	3.05×10^{-5}			

* Potentials are reported with respect to Ag/Ag^{+} in this study.

Table S6 Selected bond distances, angles and a dihedral angle of $[Ni(mnt)_2]^{2-}$

	obserbed structure in the inclusion complex **2**	calculated structure: the square-planar	calculated structure: the slightly distorted square-planar
bond distances			
Ni1–S1 / Å	2.176(3)	2.234	2.236
Ni1–S2 / Å	2.162(2)		
C1–S1 / Å	1.726(10)	1.757	1.755
C2–S2 / Å	1.720(9)		
C1–C2 / Å	1.338(11)	1.374	1.377
C1–C3 / Å	1.453(13)	1.423	1.423
C2–C4 / Å	1.435(12)		
C3–N1 / Å	1.155(17)	1.167	1.167
C4–N2 / Å	1.141(12)		
bond angles			
S1–Ni1–S2 / °	91.69(8)	91.28	91.29
S1–Ni1–S1* / °	88.25(16)	88.76	89.08
S2–Ni1–S2* / °	88.79(12)		
S1*–Ni1–S2 / °	175.08(17)	177.94	173.55
dihedral angle			
S1–S2–S2*–S1* / °	6.8(2)	2.9	9.0

* indicates the equivalent atoms generated by the symmetry operator (−x + 1, y, −z). In the case of calculated structure, almost all values except for the dihedral angle are average value.

Table S7 Coordinates of the optimized isolated state of $[Ni(mnt)_2]^{2-}$

Center Number	Atomic Number	Atomic Type	Coordinates (Angstroms) X	Y	Z
1	28	0	-0.000005	0.000025	-0.000004
2	16	0	1.562415	1.596555	-0.040495
3	6	0	3.064347	0.687282	-0.015465
4	6	0	3.064376	-0.687306	0.015352
5	6	0	4.279145	1.429009	-0.030454
6	6	0	4.279215	-1.428947	0.030460
7	7	0	5.259399	2.062146	-0.043011
8	16	0	1.562436	-1.596621	0.040079
9	7	0	5.259472	-2.062079	0.043364
10	16	0	-1.562413	1.596559	0.040488
11	6	0	-3.064345	0.687283	0.015468
12	6	0	-3.064374	-0.687306	-0.015350
13	6	0	-4.279143	1.429007	0.030460
14	6	0	-4.279213	-1.428947	-0.030451
15	7	0	-5.259399	2.062142	0.043027
16	16	0	-1.562433	-1.596623	-0.040091
17	7	0	-5.259471	-2.062076	-0.043341

Table S8 Coordinates of the optimized one-electron oxidized state of $[Ni(mnt)_2]^{2-}$

Center Number	Atomic Number	Atomic Type	Coordinates (Angstroms) X	Y	Z
1	28	0	0.000000	-0.000001	0.000000
2	16	0	1.529924	1.576919	-0.000232
3	6	0	3.025520	0.690485	-0.000030
4	6	0	3.025520	-0.690485	0.000086
5	6	0	4.237920	1.440181	-0.000196
6	6	0	4.237920	-1.440181	0.000152
7	7	0	5.217012	2.071796	-0.000544
8	16	0	1.529924	-1.576919	0.000267
9	7	0	5.217012	-2.071795	0.000522
10	16	0	-1.529924	1.576919	0.000233
11	6	0	-3.025520	0.690486	0.000030
12	6	0	-3.025520	-0.690485	-0.000085
13	6	0	-4.237920	1.440181	0.000194
14	6	0	-4.237920	-1.440181	-0.000153
15	7	0	-5.217012	2.071796	0.000542
16	16	0	-1.529924	-1.576919	-0.000266
17	7	0	-5.217013	-2.071795	-0.000523

Table S9 Coordinates of the optimized one-electron reduced state of $[Ni(mnt)_2]^{2-}$

Center Number	Atomic Number	Atomic Type	Coordinates (Angstroms) X	Y	Z
1	28	0	0.000030	-0.000003	0.000000
2	16	0	1.715904	1.651094	-0.173222
3	6	0	3.177193	0.690574	-0.072204
4	6	0	3.177194	-0.690572	0.072208
5	6	0	4.414411	1.391738	-0.145010
6	6	0	4.414411	-1.391736	0.145016
7	7	0	5.413196	1.996692	-0.205908
8	16	0	1.715904	-1.651092	0.173229
9	7	0	5.413195	-1.996693	0.205897
10	16	0	-1.715916	1.651098	0.173223
11	6	0	-3.177197	0.690572	0.072204
12	6	0	-3.177197	-0.690570	-0.072208
13	6	0	-4.414418	1.391730	0.145011
14	6	0	-4.414418	-1.391728	-0.145017
15	7	0	-5.413221	1.996654	0.205909
16	16	0	-1.715916	-1.651097	-0.173230
17	7	0	-5.413219	-1.996656	-0.205898

Table S10 Coordinates of the optimized isolated state of $\{[Ni(mnt)_2]@(\beta\text{-}CD)_2\}^{2-}$

Center Number	Atomic Number	Atomic Type	Coordinates (Angstroms) X	Y	Z
1	28	0	-0.003632	0.016929	-0.016770
2	16	0	-1.550724	1.555985	-0.502025
3	6	0	-2.938449	0.586295	-0.960526
4	6	0	-2.906668	-0.787571	-0.887234
5	6	0	-4.110963	1.247011	-1.425282
6	6	0	-4.045610	-1.547672	-1.275527
7	7	0	-5.076958	1.771471	-1.817884
8	16	0	-1.480814	-1.635043	-0.312773
9	7	0	-4.985235	-2.156952	-1.604957
10	16	0	1.415012	1.657690	0.529257
11	6	0	2.871823	0.784623	0.968193
12	6	0	2.949715	-0.583824	0.846914
13	6	0	3.985387	1.517315	1.468206
14	6	0	4.141450	-1.265938	1.221618
15	7	0	4.905308	2.099652	1.888948
16	16	0	1.601036	-1.520173	0.227604
17	7	0	5.121190	-1.814066	1.541454
18	8	0	-2.399539	7.280596	-0.054696
19	1	0	-3.010432	7.501835	0.669112
20	6	0	-2.392010	5.859538	-0.131818
21	1	0	-1.894120	5.418369	0.744488
22	1	0	-1.823906	5.590321	-1.025420
23	6	0	-3.807032	5.294214	-0.229417
24	1	0	-3.770261	4.198790	-0.210679
25	8	0	-4.475173	5.780424	0.953850
26	6	0	-5.841667	5.441276	1.062859
27	1	0	-6.184502	5.896868	1.996712

28	6	0	-6.650328	5.986062	-0.127625
29	1	0	-6.556977	7.079036	-0.121640
30	6	0	-6.058287	5.464922	-1.441843
31	1	0	-6.191992	4.373593	-1.481200
32	6	0	-4.551142	5.752296	-1.493727
33	1	0	-4.399611	6.830495	-1.627606
34	8	0	-8.028070	5.707086	0.025692
35	1	0	-8.135440	4.774074	0.305692
36	8	0	-6.723840	6.094048	-2.524772
37	1	0	-6.260500	5.831855	-3.348312
38	8	0	-4.036983	5.040503	-2.628670
39	8	0	0.470311	4.570189	-4.784470
40	1	0	0.160629	5.482017	-4.649654
41	6	0	-0.178372	3.782117	-3.792549
42	1	0	0.167626	4.049630	-2.783528
43	1	0	0.095700	2.742207	-3.985100
44	6	0	-1.694958	3.930592	-3.845903
45	1	0	-2.144013	3.338695	-3.039811
46	8	0	-1.946068	5.335989	-3.620417
47	6	0	-3.311893	5.717052	-3.621358
48	1	0	-3.313861	6.796452	-3.439869
49	6	0	-3.918248	5.391378	-4.992083
50	1	0	-3.364756	5.967345	-5.747278
51	6	0	-3.765465	3.906855	-5.303865
52	1	0	-4.339330	3.330242	-4.563095
53	6	0	-2.298039	3.495432	-5.193147
54	1	0	-1.728571	3.957044	-6.008461
55	8	0	-5.289780	5.781615	-5.005039
56	1	0	-5.692556	5.330301	-5.768124
57	8	0	-4.288914	3.707567	-6.607757
58	1	0	-4.036248	2.807067	-6.905773
59	8	0	-2.259205	2.066419	-5.314175
60	8	0	0.980988	-1.987427	-4.689828

61	1	0	0.607283	-2.864843	-4.486593
62	6	0	0.198317	-1.034822	-3.974295
63	1	0	0.862952	-0.227127	-3.650321
64	1	0	-0.235400	-1.483001	-3.074782
65	6	0	-0.932482	-0.405532	-4.795788
66	1	0	-1.678785	-0.010154	-4.095051
67	8	0	-0.389814	0.692523	-5.563623
68	6	0	-1.360156	1.479703	-6.225338
69	1	0	-0.797528	2.250630	-6.759765
70	6	0	-2.144756	0.603311	-7.219065
71	1	0	-1.430344	0.276178	-7.989832
72	6	0	-2.715775	-0.634527	-6.524480
73	1	0	-3.479567	-0.324832	-5.804951
74	6	0	-1.603395	-1.375783	-5.781842
75	1	0	-0.837359	-1.718254	-6.488220
76	8	0	-3.173879	1.380170	-7.821961
77	1	0	-3.774110	0.753243	-8.263916
78	8	0	-3.420725	-1.459541	-7.460188
79	1	0	-2.797067	-1.763770	-8.144476
80	8	0	-2.181836	-2.499499	-5.114589
81	8	0	0.056181	-6.229982	-2.597752
82	1	0	0.364507	-6.010037	-3.493540
83	6	0	-0.554403	-5.049954	-2.085565
84	1	0	0.195144	-4.273803	-1.875887
85	1	0	-1.036412	-5.323712	-1.144766
86	6	0	-1.596095	-4.485145	-3.043677
87	1	0	-1.963888	-3.528798	-2.656480
88	8	0	-0.879561	-4.259518	-4.287889
89	6	0	-1.652985	-3.782378	-5.378239
90	1	0	-0.964107	-3.747195	-6.226916
91	6	0	-2.822424	-4.727606	-5.669299
92	1	0	-2.409445	-5.697884	-5.967322
93	6	0	-2.798760	-5.427598	-3.257686

94	1	0	-2.439718	-6.441202	-3.475324
95	8	0	-3.619027	-4.279576	-6.752738
96	1	0	-3.707462	-3.304491	-6.695063
97	8	0	-4.699729	-5.871887	-4.648972
98	1	0	-5.175181	-5.559720	-5.439307
99	8	0	-3.604945	-5.424527	-2.080406
100	8	0	-3.273332	-6.547397	2.851744
101	1	0	-2.806510	-6.519587	3.701072
102	6	0	-2.823699	-5.433772	2.079956
103	1	0	-1.755284	-5.530559	1.847639
104	1	0	-2.984163	-4.491481	2.618347
105	6	0	-3.597883	-5.379952	0.770563
106	1	0	-3.363885	-4.429433	0.275778
107	8	0	-3.122588	-6.469569	-0.045924
108	6	0	-3.758960	-6.598005	-1.297160
109	1	0	-3.280110	-7.450094	-1.790005
110	6	0	-5.267501	-6.847686	-1.127711
111	1	0	-5.389030	-7.765285	-0.538925
112	6	0	-5.889691	-5.688899	-0.338723
113	1	0	-5.809655	-4.770132	-0.937694
114	6	0	-5.120068	-5.475605	0.970917
115	1	0	-5.341884	-6.312079	1.641602
116	8	0	-5.896466	-7.088549	-2.370375
117	1	0	-5.550676	-6.455161	-3.034000
118	8	0	-7.249162	-5.998575	-0.072220
119	1	0	-7.581987	-5.325069	0.556141
120	8	0	-5.608828	-4.250615	1.547020
121	8	0	-4.699015	-1.545673	5.693177
122	1	0	-4.808100	-2.505175	5.806752
123	6	0	-4.231305	-1.359789	4.360540
124	1	0	-3.225070	-1.782872	4.233720
125	1	0	-4.186321	-0.281588	4.189839
126	6	0	-5.169557	-2.000310	3.341412

127	1	0	-4.765199	-1.866827	2.329908
128	8	0	-5.185751	-3.403331	3.683853
129	6	0	-6.014901	-4.236710	2.892669
130	1	0	-5.921473	-5.233231	3.334385
131	6	0	-7.465363	-3.726585	2.969619
132	1	0	-7.805695	-3.804864	4.008553
133	6	0	-7.537046	-2.264506	2.538644
134	1	0	-7.229187	-2.184730	1.486916
135	6	0	-6.598063	-1.426694	3.399698
136	1	0	-6.942866	-1.451446	4.440021
137	8	0	-8.348298	-4.458487	2.113388
138	1	0	-8.692068	-5.225411	2.600085
139	8	0	-8.862222	-1.763955	2.700455
140	1	0	-9.448857	-2.352052	2.193600
141	8	0	-6.620285	-0.081823	2.911233
142	8	0	-4.801212	4.309489	4.611790
143	1	0	-5.252478	3.714163	5.234375
144	6	0	-4.271734	3.473645	3.588013
145	1	0	-3.468092	2.834293	3.976724
146	1	0	-3.849947	4.134012	2.826971
147	6	0	-5.355379	2.597713	2.965056
148	1	0	-4.909937	1.937178	2.209643
149	8	0	-5.863364	1.810168	4.066618
150	6	0	-6.962922	0.962559	3.806787
151	1	0	-7.242868	0.546903	4.779137
152	6	0	-8.123815	1.769391	3.207202
153	1	0	-8.421683	2.518355	3.951232
154	6	0	-7.651416	2.512249	1.954486
155	1	0	-7.322591	1.784226	1.198651
156	6	0	-6.483292	3.420322	2.310648
157	1	0	-6.821807	4.186862	3.017899
158	8	0	-9.277101	0.995760	2.936453
159	1	0	-9.011577	0.097170	2.639618

160	8	0	-8.711668	3.308847	1.432958
161	1	0	-9.483969	2.718395	1.371207
162	8	0	-6.016791	4.035728	1.108473
163	6	0	-3.663458	-4.923129	-4.407314
164	1	0	-4.100711	-3.958836	-4.112626
165	8	0	1.376712	7.199042	0.438127
166	1	0	1.937778	7.563714	-0.267697
167	6	0	1.622050	5.797210	0.441601
168	1	0	1.217307	5.321817	-0.464348
169	1	0	1.104744	5.384143	1.310253
170	6	0	3.112420	5.482760	0.529723
171	1	0	3.262403	4.399658	0.455944
172	8	0	3.693086	6.136940	-0.618573
173	6	0	5.094793	6.015199	-0.738502
174	1	0	5.362564	6.570972	-1.642157
175	6	0	5.816636	6.603740	0.486977
176	1	0	5.572339	7.671882	0.537545
177	6	0	5.302630	5.936974	1.768410
178	1	0	5.595697	4.876262	1.755023
179	6	0	3.769466	5.996855	1.819555
180	1	0	3.458458	7.034535	1.992272
181	8	0	7.220384	6.526766	0.330499
182	1	0	7.454472	5.635291	-0.002561
183	8	0	5.865537	6.601373	2.887701
184	1	0	5.436139	6.240332	3.692073
185	8	0	3.364608	5.174011	2.923599
186	8	0	-1.041398	3.937280	4.998786
187	1	0	-0.889733	4.892488	4.899229
188	6	0	-0.270624	3.304938	3.982449
189	1	0	-0.647821	3.558915	2.981453
190	1	0	-0.378671	2.227870	4.128162
191	6	0	1.203940	3.682826	4.065347
192	1	0	1.746987	3.196823	3.246598

193	8	0	1.242794	5.117158	3.894393
194	6	0	2.537037	5.696358	3.929878
195	1	0	2.380321	6.770170	3.788133
196	6	0	3.175142	5.411910	5.295801
197	1	0	2.537363	5.870912	6.064569
198	6	0	3.240860	3.910250	5.550698
199	1	0	3.898206	3.452955	4.796106
200	6	0	1.851264	3.293294	5.405522
201	1	0	1.212380	3.635805	6.227766
202	8	0	4.473605	5.999788	5.339190
203	1	0	4.933057	5.586769	6.091882
204	8	0	3.777675	3.736264	6.852713
205	1	0	3.673987	2.792545	7.101756
206	8	0	2.023301	1.871212	5.475402
207	8	0	-0.679038	-2.515124	4.627478
208	1	0	-0.199937	-3.323647	4.367451
209	6	0	-0.032224	-1.429930	3.967801
210	1	0	-0.797928	-0.693959	3.701017
211	1	0	0.439480	-1.764090	3.038171
212	6	0	1.020584	-0.711657	4.818250
213	1	0	1.695155	-0.175934	4.139001
214	8	0	0.350984	0.253612	5.660993
215	6	0	1.222748	1.122459	6.358508
216	1	0	0.574064	1.780984	6.943414
217	6	0	2.136927	0.302222	7.287117
218	1	0	1.490309	-0.164841	8.045412
219	6	0	2.853585	-0.802306	6.507524
220	1	0	3.552851	-0.346140	5.800583
221	6	0	1.833924	-1.640219	5.734406
222	1	0	1.135816	-2.122395	6.429339
223	8	0	3.064268	1.175033	7.923252
224	1	0	3.751215	0.611349	8.321738
225	8	0	3.680734	-1.582138	7.378890

226	1	0	3.115593	-2.003568	8.051750
227	8	0	2.541966	-2.636528	4.993768
228	8	0	0.725191	-6.443045	2.260895
229	1	0	0.401152	-6.317391	3.169158
230	6	0	1.185971	-5.169681	1.821875
231	1	0	0.347850	-4.474837	1.671122
232	1	0	1.681366	-5.324114	0.860792
233	6	0	2.170217	-4.547453	2.805057
234	1	0	2.414613	-3.530840	2.478725
235	8	0	1.454621	-4.490871	4.068596
236	6	0	2.183207	-3.990079	5.179041
237	1	0	1.512532	-4.095852	6.036268
238	6	0	3.467729	-4.795750	5.395320
239	1	0	3.186633	-5.826462	5.639180
240	6	0	3.481203	-5.348525	2.945777
241	1	0	3.250285	-6.409183	3.105476
242	8	0	4.223534	-4.314310	6.493839
243	1	0	4.187632	-3.334353	6.492524
244	8	0	5.450828	-5.632889	4.284817
245	1	0	5.900554	-5.308255	5.085030
246	8	0	4.260807	-5.177612	1.763770
247	8	0	3.997780	-5.960084	-3.234206
248	1	0	3.533483	-5.901892	-4.083332
249	6	0	3.453511	-4.954214	-2.379160
250	1	0	2.399647	-5.165156	-2.156288
251	1	0	3.526386	-3.962847	-2.843340
252	6	0	4.229262	-4.927775	-1.070431
253	1	0	3.907726	-4.046615	-0.501872
254	8	0	3.874394	-6.119066	-0.339053
255	6	0	4.530164	-6.270457	0.900026
256	1	0	4.146100	-7.200922	1.330272
257	6	0	6.055630	-6.349632	0.713314
258	1	0	6.267347	-7.205175	0.060189

259	6	0	6.548878	-5.078698	0.009709
260	1	0	6.377216	-4.217749	0.671955
261	6	0	5.751040	-4.856676	-1.281078
262	1	0	6.048780	-5.620835	-2.006476
263	8	0	6.713758	-6.613985	1.935898
264	1	0	6.323732	-6.051460	2.637914
265	8	0	7.931057	-5.223340	-0.277940
266	1	0	8.188373	-4.469470	-0.847931
267	8	0	6.107939	-3.553543	-1.776307
268	8	0	4.928926	-0.755437	-5.790887
269	1	0	5.155237	-1.687735	-5.949074
270	6	0	4.420322	-0.696897	-4.460937
271	1	0	3.467517	-1.237779	-4.378300
272	1	0	4.246861	0.358259	-4.236838
273	6	0	5.412746	-1.275522	-3.456032
274	1	0	4.979690	-1.247526	-2.447798
275	8	0	5.604937	-2.645251	-3.871596
276	6	0	6.521853	-3.413889	-3.112324
277	1	0	6.555904	-4.387893	-3.609153
278	6	0	7.899677	-2.726401	-3.135806
279	1	0	8.257783	-2.701823	-4.171432
280	6	0	7.785064	-1.294262	-2.621511
281	1	0	7.455026	-1.314599	-1.573857
282	6	0	6.761288	-0.530404	-3.453857
283	1	0	7.119689	-0.454181	-4.487044
284	8	0	8.857677	-3.395167	-2.308568
285	1	0	9.310952	-4.070403	-2.839200
286	8	0	9.039566	-0.625442	-2.727228
287	1	0	9.687231	-1.164452	-2.240559
288	8	0	6.612969	0.777545	-2.892833
289	8	0	4.229050	4.972616	-4.356237
290	1	0	4.762881	4.485335	-5.006868
291	6	0	3.820565	4.011643	-3.389028

292	1	0	3.117487	3.286907	-3.822637
293	1	0	3.307810	4.558166	-2.594521
294	6	0	5.015346	3.258540	-2.809989
295	1	0	4.666637	2.496911	-2.100817
296	8	0	5.628554	2.619146	-3.953043
297	6	0	6.825530	1.903838	-3.726679
298	1	0	7.160516	1.580859	-4.716729
299	6	0	7.871374	2.816467	-3.069627
300	1	0	8.077704	3.635963	-3.768928
301	6	0	7.301116	3.424172	-1.785044
302	1	0	7.070928	2.619841	-1.070927
303	6	0	6.021113	4.185679	-2.098676
304	1	0	6.251634	5.032914	-2.755412
305	8	0	9.110854	2.179027	-2.825490
306	1	0	8.956382	1.241319	-2.575263
307	8	0	8.242917	4.327550	-1.212226
308	1	0	9.086289	3.842246	-1.169045
309	8	0	5.480206	4.657773	-0.863306
310	6	0	4.299919	-4.808792	4.113251
311	1	0	4.606804	-3.781699	3.870370
